# Optimizing community case management strategies to achieve equitable reduction of childhood pneumonia mortality: An application of Equitable Impact Sensitive Tool (EQUIST) in five low– and middle–income countries

**DOI:** 10.7189/jogh.02.020402

**Published:** 2012-12

**Authors:** Donald Waters, Evropi Theodoratou, Harry Campbell, Igor Rudan, Mickey Chopra

**Affiliations:** 1 Centre for Population Health Sciences and Global Health Academy, The University of Edinburgh Medical School, Edinburgh, Scotland, UK; 2 UNICEF, New York, USA; * Equal authors’ contribution

## Abstract

**Background:**

The aim of this study was to populate the Equitable Impact Sensitive Tool (EQUIST) framework with all necessary data and conduct the first implementation of EQUIST in studying cost–effectiveness of community case management of childhood pneumonia in 5 low– and middle–income countries with relation to equity impact.

**Methods:**

Wealth quintile–specific data were gathered or modelled for all contributory determinants of the EQUIST framework, namely: under–five mortality rate, cost of intervention, intervention effectiveness, current coverage of intervention and relative disease distribution. These were then combined statistically to calculate the final outcome of the EQUIST model for community case management of childhood pneumonia: US$ per life saved, in several different approaches to scaling–up.

**Results:**

The current ‘mainstream’ approach to scaling–up of interventions is never the most cost–effective. Community–case management appears to strongly support an ‘equity–promoting’ approach to scaling–up, displaying the highest levels of cost–effectiveness in interventions targeted at the poorest quintile of each study country, although absolute cost differences vary by context.

**Conclusions:**

The relationship between cost–effectiveness and equity impact is complex, with many determinants to consider. One important way to increase intervention cost–effectiveness in poorer quintiles is to improve the efficiency and quality of delivery. More data are needed in all areas to increase the accuracy of EQUIST–based estimates.

Three years are left until the 2015 deadline to achieve the Millennium Development Goals (MDGs) and much progress has been achieved. A recent Inter–Agency Group for Child Mortality Estimation (IGME) meeting reported a child mortality decrease of over one third from 1990 to 2010 [1]. However, an unforeseen issue is that in many low– and middle–income countries (LMICs) a decrease in under–five mortality rate (U5MR) has been accompanied by increased inequity in health outcomes between the poor and those better off [2]. This important consideration has been discussed extensively in a recent United Nations Children’s Fund (UNICEF) report which argues for abandoning the ‘mainstream approach’ where scaling–up of child health interventions is first provided to more readily accessible (and typically wealthier) groups in society. Instead, an ‘equity–focused’ approach is suggested, contending that it is more cost–effective to target interventions at the poorest in society, resulting in a greater U5MR decrease while also positively impacting upon equity.

To test this hypothesis, a tool is required that can address the many determinants in the multifaceted relationship between cost–effectiveness and equitable impact in child mortality reduction. Although a number of tools have been developed to assist intervention prioritization at local and national levels – such as Marginal Budgeting for Bottlenecks (MBB, supported by UNICEF) [3],Choice of Interventions that are Cost–Effective (CHOICE, promoted by the World Health Organization – WHO) [4], and the Lives Saved Tool (LiST, developed by Johns Hopkins University and Futures Institute) [5], none of these tools can fully address equitable impact considerations as they make no allowance for income–related inequalities in countries.

We recently described a conceptual framework that helps understanding the complex interplay between determinants of cost–effectiveness and equitable impact in child mortality reduction (see **Figure 1** for visual representation of the framework), also exposing the importance of several critical determinants for which information is typically lacking [6]. The tool based on this framework has been named EQUIST – **EQU**itable **I**mpact **S**ensitive **T**ool [7]. This study presents the first implementation of EQUIST to test the hypothesis that, against conventional wisdom and prevailing practices, significantly higher gains in child mortality reduction can be achieved through an equity–focused approach to scaling–up of child health interventions without compromising cost–effectiveness.

**Figure 1:**
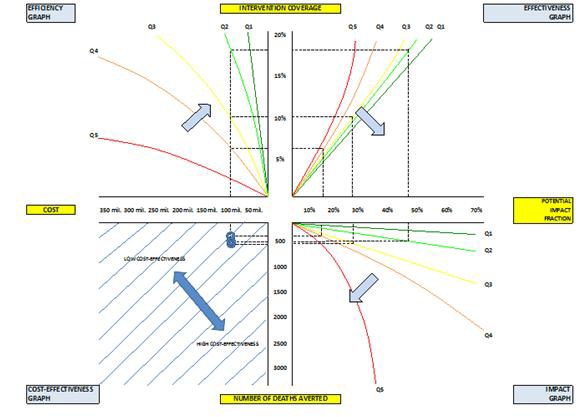
Conceptual framework for EQUIST [6], demonstrating a hypothetical planning exercise assessing the cost–effectiveness of delivery of a new intervention to different equity strata in the population (quintile Q2 vs Q3 vs Q4) with a fixed budget.

## METHODS

**Figure 2:**
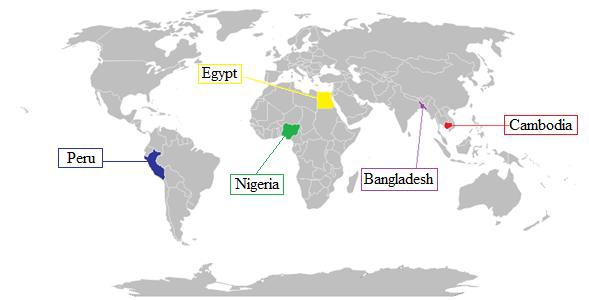
Exemplar countries used in the study.

To test EQUIST, five exemplar countries representative of larger WHO regions were used: Nigeria (Sub–Saharan Africa), Egypt (Eastern Mediterranean), Bangladesh (South–East Asia), Cambodia (Western Pacific) and Peru (Americas) (**Figure 2****)**. These were selected because of their large size and relatively adequate information reported by equity strata. It was also decided to focus on a single disease – pneumonia, which is still the leading cause of child deaths globally [8]. To allow appropriate close scrutiny, a single intervention was studied, namely community case management with antibiotics (CCM), which has proven efficacy in reducing child pneumonia mortality [9,10]. The Child Health Epidemiology Reference Group (CHERG) estimates of worldwide child mortality for 2008 [11] were used, as these data are complete, high–quality, and coincide closely with the most recent Demographic and Health Survey (DHS) data in the five chosen countries [12-16].

### Estimates of U5MR

The first step in populating this model was to establish U5MR distribution by wealth quintiles in the five countries, along with the quintile ratio (QR), a commonly used measure of equity (the closer QR is to 1, the closer the country is to health outcomes equity [17]). For all of the countries, data were available from DHS reports 2007, 2008 or 2010 [12-16], therefore correlating strongly with the most recent CHERG data.

### Cost estimates

The second step was to estimate the cost of scaling–up CCM in each quintile from its existing level of coverage. For more accurate estimation, cost was split for CCM into antibiotic costs and non–antibiotic costs. It was assumed that the direct costs of antibiotics (ie, the medicines themselves) would be constant across countries and quintiles, while the non–antibiotic costs were likely to be different due to factors including geography, infrastructure and human resources [18].

Direct antibiotic costs for CCM were taken as US$ 0.27 for all quintiles in all areas [8]. Non–antibiotic costs were modelled based on an unpublished report from Pakistan [19], which was the only available source, highlighting the general scarcity of information on this important variable. Direct CCM cost was added to non–CCM costs calculated from the quintile’s U5MR to obtain an estimate of the cost for each intervention per child treated in any individual quintile. In the next step, to gain a more accurate measure of the cost of treatment per quintile, the cost per child treated was multiplied by the total number of under–five episodes of pneumonia in each quintile. The number of episodes was estimated by combining a modelled case fatality rate (CFR) for each quintile with the estimated number of under–five pneumonia deaths.

### Estimates of current intervention coverage

The third step was to determine coverage levels of the chosen intervention in the five countries in 2008. Coverage with CCM was assumed to be the same as the indicator “% under–fives with suspected pneumonia receiving antibiotics” used in UNICEF’s The State of the World’s Children (SOWC) reports.

### Effectiveness estimates

The fourth step was to estimate how CCM’s effectiveness varied according to the quintile in which it was implemented and therefore calculate the quintile–specific potential impact fraction (PIF). Effectiveness was modelled by graphing effectiveness reported in each study used in a review of CCM [9], against the U5MR for the specific country at the year of study publication (taken from Child Mortality Estimates database [20]). The estimate for each quintile given using the equation of this graph was then adjusted upwards by 50% of the remaining effectiveness gap as suggested in the methods used by Theodoratou et al [9] and the LiST[5].

### Disease proportion estimates

Finally, it was necessary to populate the model with disease burden estimates for each disease in each quintile. This was initially attempted through systematic literature review; however an attempt (using MEDLINE, EMBASE and Global Health databases) yielded insufficient data therefore it was decided to model them instead. Data on distributions of under–five mortality deaths by cause for all countries (from the CHERG report [11]) were combined with U5MR data for each country (from SOWC 2009 [21]) in a model, resulting in estimates of cause–specific mortality in each quintile for each global region, and subsequently for the exemplar countries.

### Final model

Once the model was fully populated with data necessary to evaluate cost–effectiveness and impact on mortality and equity of community case management for under–five pneumonia mortality, it was decided to compare the cost per number of lives saved for scaling–up the intervention in the next wealthiest 10% of the uncovered population (‘inequity promoting’ approach [6]), in the middle 10% of the uncovered population (‘equity neutral’ [6]), in the poorest 10% of the population (‘equity–promoting’ [6]), and finally a 10% scale–up in the ‘mainstream approach’ (coverage scale–up continuing to follow current quintile–specific relative distribution [22]).

Further detailed information on the methods described above in each section is presented in Online Supplementary Document(Online Supplementary Document) (table w1).

## RESULTS

**Figure 3:**
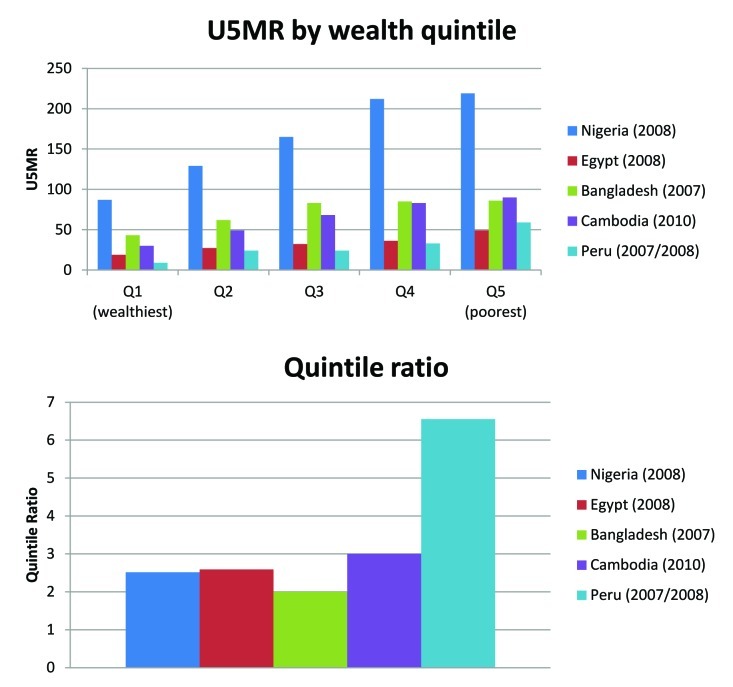
U5MR and inequity by wealth quintiles in exemplar countries.

**Figure 4:**
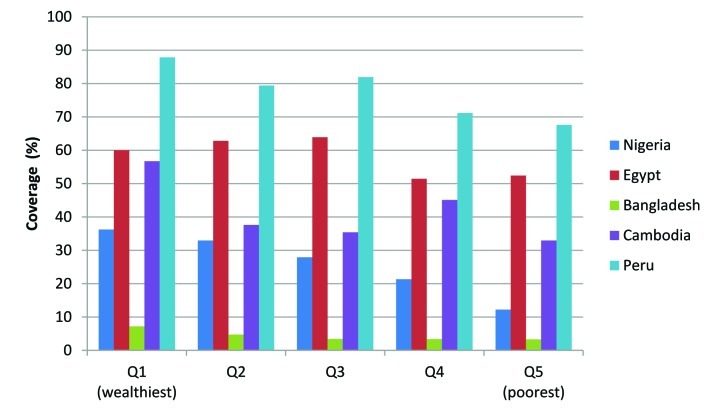
Community case management (CCM) coverage estimates by wealth quintiles in exemplar countries.

**Table 1** and **Figure 3** show the estimates of U5MR (as deaths per 1000 live births) by quintile for the exemplar countries. Quintile ratios for each country are shown in **Figure 4**. Data for each country exhibit expected trends of U5MR decreasing with wealth [11]; however, not all to similar degrees. Nigeria is shown to have a noticeably higher U5MR than the other 5 countries and this is supported by the 2008 CHERG report, which found the significant majority of under–5 mortality to occur in Africa [11]. Peru has the greatest QR ratio, suggesting it has the highest inequity. Bangladesh exhibits a higher U5MR in each quintile than Peru but a much less significant U5MR variation between quintiles (especially quintile Q3–Q5), and the lowest QR of the five countries, suggesting it is the most equitable studied.

**Table 1:** Cost of community case management (CCM) per child treated in each country by wealth quintile (Q)

	Overall	Q1 (wealthiest)	Q2	Q3	Q4	Q5 (poorest)
**Nigeria:**						
U5MR	189.00	87.00	129.00	165.00	212.00	219.00
Cost of antibiotic (US$)	0.27	0.27	0.27	0.27	0.27	0.27
Non–antibiotic cost (US$)	2.52	1.43	1.87	2.26	2.76	2.84
Total cost of CCM per child treated	2.79	1.70	2.14	2.53	3.03	3.11
**Egypt:**						
U5MR	36.00	18.90	27.20	32.20	36.10	49.00
Cost of antibiotic (US$)	0.27	0.27	0.27	0.27	0.27	0.27
Non–antibiotic cost (US$)	0.88	0.70	0.79	0.84	0.88	1.02
Total cost of CCM per child treated	1.15	0.97	1.06	1.11	1.15	1.29
**Bangladesh:**						
U5MR	61.00	43.00	62.00	83.00	85.00	86.00
Cost of antibiotic (US$)	0.27	0.27	0.27	0.27	0.27	0.27
Non–antibiotic cost (US$)	1.15	0.95	1.16	1.38	1.40	1.41
Total cost of CCM per child treated	1.42	1.22	1.43	1.65	1.67	1.68
**Cambodia:**						
U5MR	54.00	30.00	49.00	68.00	83.00	90.00
Cost of antibiotic (US$)	0.27	0.27	0.27	0.27	0.27	0.27
Non–antibiotic cost (US$)	1.07	0.82	1.02	1.22	1.38	1.46
Total cost of CCM per child treated	1.34	1.09	1.29	1.49	1.65	1.73
**Peru:**						
U5MR	27.00	9.00	24.00	24.00	33.00	59.00
Cost of antibiotic (US$)	0.27	0.27	0.27	0.27	0.27	0.27
Non–antibiotic cost (US$)	0.78	0.59	0.75	0.75	0.85	1.13
Total cost of CCM per child treated	1.05	0.86	1.02	1.02	1.12	1.40

U5MR – under–five mortality rate

**Figure 4 **shows estimates of coverage by wealth quintile for community case management (CCM). Although the estimates of coverage by quintile for community case management generally follow expected trends of decreasing coverage with increasing poverty (the greatest differences by quintile being found in Nigeria and Cambodia), Egypt exhibits a slightly unexpected pattern with increased CCM of suspected pneumonia in Q2 and Q3 as compared with Q1. This is thought to be due to the fact that in rich urban communities (ie, Q1), medical professionals are trying to avoid over–treating (and therefore promoting antibiotic resistance), but in poorer quintiles this is not the case and more cases are treated aggressively with antibiotics, explaining the higher coverage levels. There is then a dip again in coverage observed in Q4 and Q5 in Egypt, likely to be explained by poor access to health care in the poorest part of the population.

**Figure 5:**
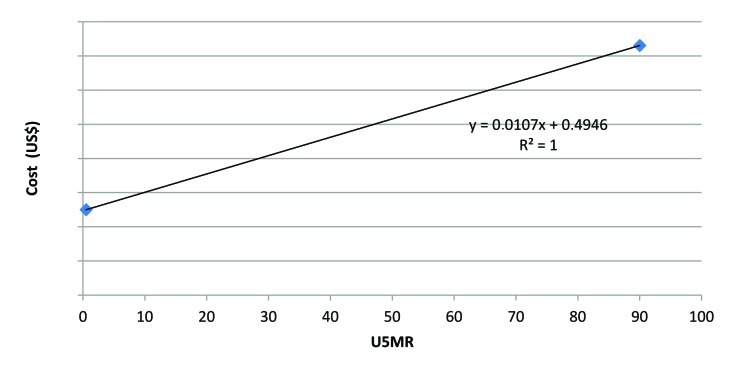
Non–antibiotic cost estimate model.

**Figure 5** illustrates the model for non–antibiotic cost for CCM and **Table 1** shows the final ‘cost per child treated’ calculated for CCM in each quintile of the exemplar countries. A consistent trend is observed of increasing intervention cost from Q1–Q5 with Cambodia showing the biggest cost differences between Q1–Q2 and Q2–Q3 and Nigeria between Q3 and Q4. These countries also show the highest overall cost difference.

**Figure 6:**
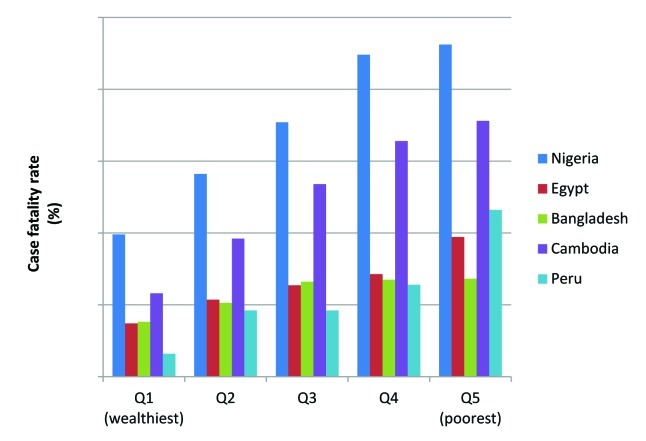
Case fatality rates by wealth quintile**s** in exemplar countries.

**Figure 7:**
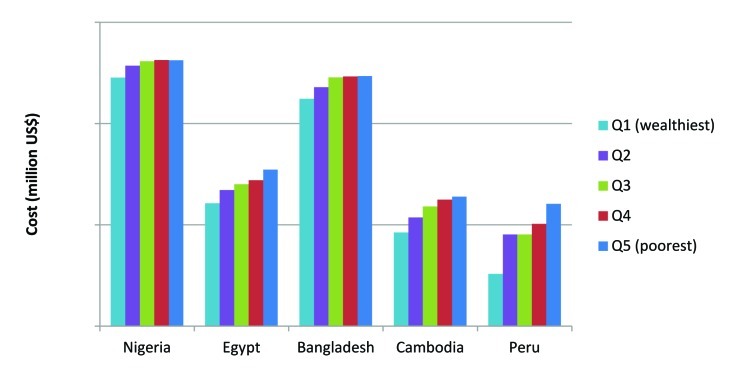
Cost of community case management (CCM) treatment by wealth quintile in exemplar countries.

**Figure 6** illustrates the case fatality rates modelled for each quintile (region–specific graphic models are displayed in the Online Supplementary Document(Online Supplementary Document), table w2) and **Figure 7** shows the resulting adjusted cost per quintile treated. Again the trend almost uniformly shows an increasing cost from Q1–Q5, with one noticeable difference – the finding of Q5 in Nigeria being marginally less costly than Q4, suggesting that the case fatality in Q5 is so high that scaling–up in this quintile will save more money than in Q4 for the same investment.

**Figure 8:**
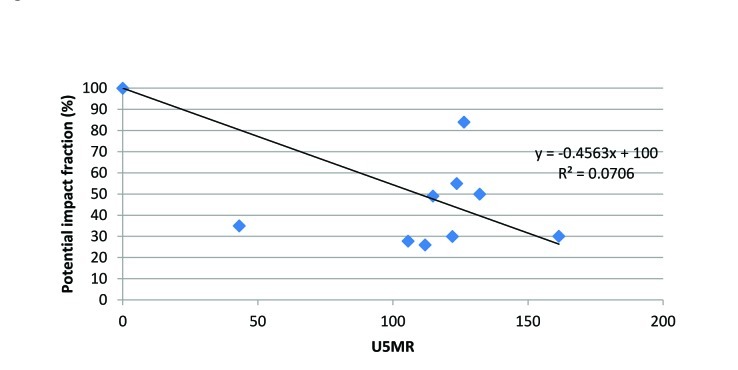
Effectiveness/Potential Impact Fraction (PIF) model for community case management (CCM).

**Figure 9:**
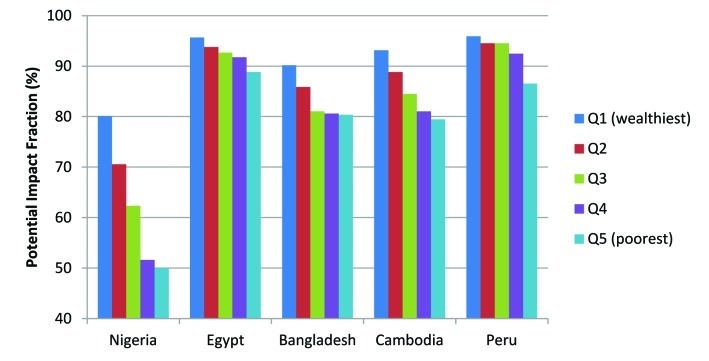
Effectiveness/Potential Impact Fraction (PIF) model for community case management (CCM) in each country by wealth quintile**s** in exemplar countries.

**Table 2** shows data used to model CCM effectiveness/PIF and **Figure 8** illustrates the model. Importantly, **Table 2**highlights scarcity in CCM effectiveness data, as although these papers were carefully screened in a recent review and found to be high–quality [9], none of them were published after 1998. **Figure 9** illustrates the upwards–adjusted effectiveness data for each quintile in each country, showing a continual trend of decreasing effectiveness from Q1 to Q5 but with the biggest decrease being seen in Nigeria, where the poorer quintiles have a significantly higher U5MR.

**Table 2:** Data for CCM effectiveness/Potential Impact Fraction (PIF) modelling

Study	Location	Year	U5MR	Effectiveness
Mtango et al [23]	Tanzania	1986	161.40	30.10
Pandey et al [24]	Nepal	1989	126.30	84.00
Bang et al [25]	India	1990	114.80	49.10
Khan et al [26]	Pakistan	1990	123.60	55.00
Reddaiah et al [27]	India	1991	111.90	26.00
Pandey et al [28]	Nepal	1991	122.00	30.00
Fauveau et al [29]	Bangladesh	1992	132.10	50.00
Agarwal et al [30]	India	1993	105.60	27.80
WHO [31]	Philippines	1998	43.10	35.00
Our suggestion [6]	Global	2012	0.00	100.00

U5MR – under–five mortality rate, WHO – World Health Organization

**Figure 10:**
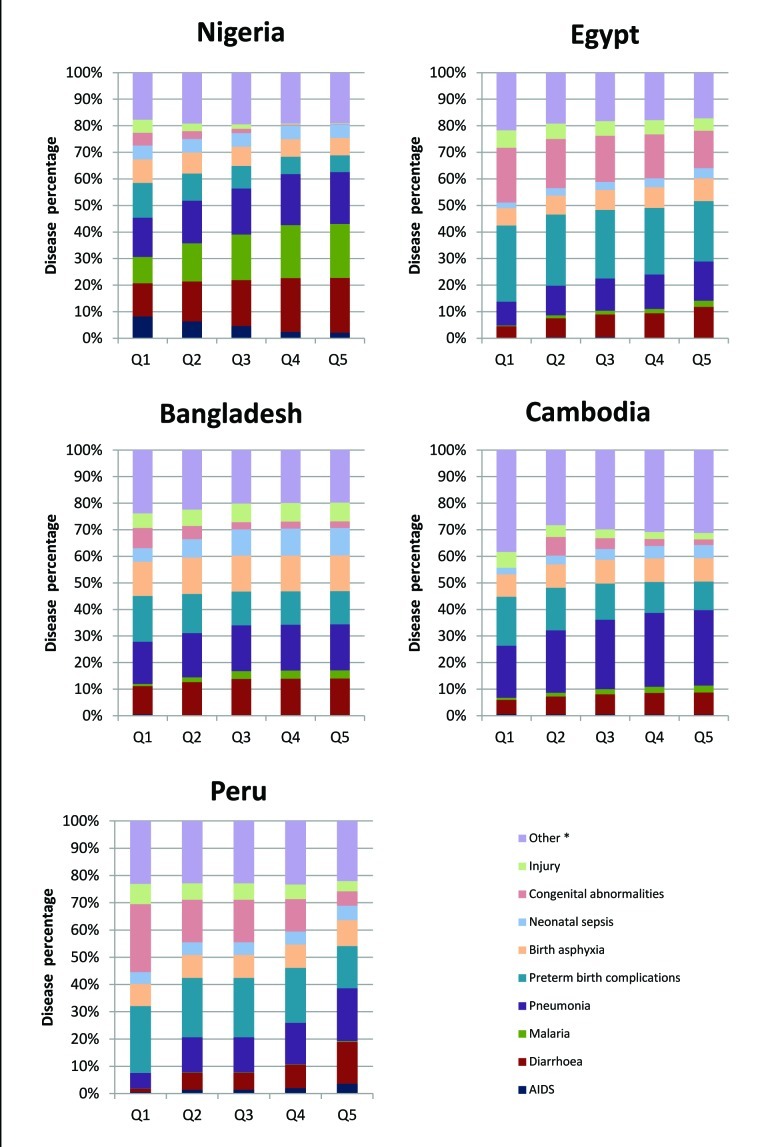
Modelled cause–specific child mortality by wealth quintiles in exemplar countries.

**Figure 10** shows quintile–specific disease proportion estimates for each of the exemplar countries, expressed as the percentage of the total under–five mortality burden. Significant differences across wealth quintiles in causes of death in those aged under–five can be seen in each of the five exemplar countries with all studied countries showing increasing proportions of deaths due to malaria, pneumonia and diarrhoea in poorer quintiles while proportions of deaths due to congenital abnormalities, preterm birth complications and injury decrease as poverty increases. This is thought to be due to the fact that infectious diseases such as malaria and pneumonia are treated more effectively in richer populations resulting in a diminished proportion of deaths due to these causes but an increased proportion of deaths due to causes that even well–funded health systems struggle to deal with such as congenital abnormalities or injury.

Bangladesh shows an interesting pattern of birth asphyxia, which takes up highest proportion of mortality in Q2 and Q3, potentially suggesting that in these quintiles although the infectious diseases which are prevalent in poorer quintiles are still well treated, the health care facilities in these quintiles are not as good as in Q1 and so more babies die of birth asphyxia.

WHO region–specific disease proportion estimates and models for disease proportion against U5MR and quintile–specific numbers of deaths in 2008 from each of these causes of death in each of the five exemplar countries are presented in the Online Supplementary Document(Online Supplementary Document) (tables w3 and w4).

**Figure 11:**
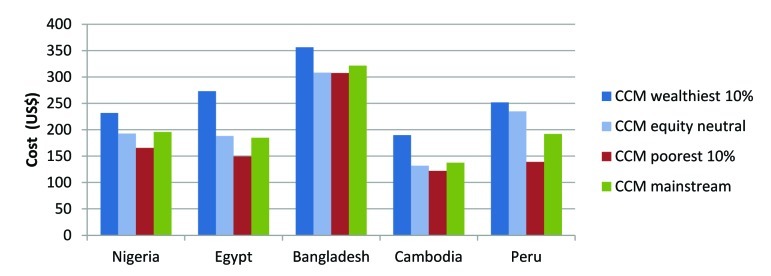
Estimated cost per life saved in exemplar countries.

**Figure 11** illustrates the final results: cost per life saved (in US$) for each quintile in each country by scaling–up CCM in the different studied strategies.

Strikingly, the ‘mainstream’ approach for CCM in all countries is not the most cost–effective, instead an equity–promoting approach always delivers the greatest cost–effectiveness in terms of US$ per life saved. The absolute cost differences between this and the next most costly approach differ with context, varying from US$ 59.92 per life saved in Peru to US$ 1.10 in Bangladesh, where an equity–promoting approach is of almost the same cost–effectiveness as an equity–neutral approach of scaling up in the middle uncovered 10%. It is thought this is due to the differences in U5MR from Q3 to Q5 being relatively small in Bangladesh, resulting in the differences in disease burden, coverage, effectiveness and cost also not being large. This can be contrasted with Peru where the greatest difference in cost–effectiveness is between equity–promoting and equity–neutral and the greatest difference in U5MR is between Q5–Q3. This potentially suggests that in more inequitable contexts such as Peru (which has the highest QR of the countries studied), an equity–promoting approach will have a greater impact when compared with more equitable contexts. Egypt is the only modelled country where the next most cost–effective scale–up option is the ‘mainstream’ approach, possibly due to an already relatively equitable coverage of CCM across quintiles (a difference of only 7.6% coverage from Q1 to Q5).

Nigeria is an interesting context to study as due to its exceedingly high U5MR in poorer quintiles, the effectiveness modelled for Q5 is 50.4%. It was thought that this might result in an equity–promoting scale–up delivering poor results however what is observed in actuality is that scale–up in Q5 is still the most cost–effective. This highlights that the childhood pneumonia burden in this stratum is so great that even treating 50% will result in a huge improvement, but also that any intervention which could improve effectiveness of CCM could further enhance this and result in extremely significant reductions in Nigeria’s overall childhood pneumonia burden.

**Figure 12:**
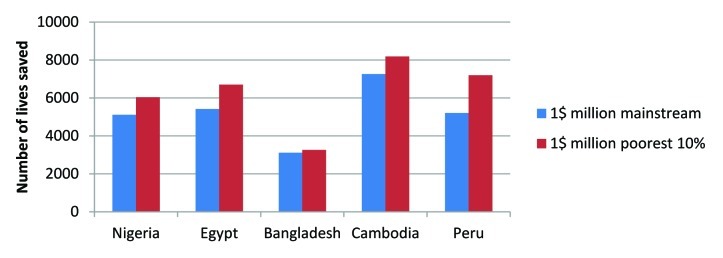
Estimated number of lives saved in Mainstream vs Equity–promoting models for the same investment.

**Table 3** shows the exact numbers of lives saved from the same investment of US$ 1 000 000 either in the ‘mainstream’ approach or an equity–promoting approach with targeted CCM scaling up in Q5. This is illustrated in **Figure 12**. Although it can be seen that an equity–promoting approach to investment in CCM always results in a greater saving of life than the ‘mainstream’ approach, the gradient of the difference varies significantly between countries with the greatest contrast found in Peru, the country with the highest QR and therefore greatest inequity, again suggesting that an equity–promoting approach is potentially most valuable in countries with the highest inequity.

**Table 3:** Number of lives saved for US$ 1 million investment according to Mainstream vs Equity–promoting model

Country	Mainstream model	Equity–promoting (Poorest 10%) model	Mainstream vs Equity– promoting (Poorest 10%) model
Nigeria	5108	6037	929
Egypt	5411	6698	1287
Bangladesh	3110	3254	144
Cambodia	7262	8188	925
Peru	5209	7191	1982

The results for all aspects of this study are further explained the Online Supplementary Document(Online Supplementary Document) (table w1).

## DISCUSSION

This study aimed to populate EQUIST [6] with real data from five exemplar LMICs and thereby investigate cost–effectiveness of different strategies to scaling–up childhood pneumonia interventions. Apart from noting the scarcity of high–quality information in this area, this paper has delivered three major outcomes. Firstly, the information generated through modelling to populate EQUIST represents a novel contribution to understanding equity and child health in LMICs. Secondly, this paper has shown that EQUIST is potentially a valuable tool for evaluating cost–effectiveness of different approaches to scaling–up health interventions. Finally, this first implementation of EQUIST has highlighted the complexity of relations between the multiple determinants of cost–effectiveness and equitable impact in LMIC child mortality reduction. Unexpected patterns are seen both in each variable’s distributions and in the final outcome results, further compounded by the difficulty in determining which of the multiple contributory variables is influencing the results most. This emphasizes that data on equity and cost–effectiveness for intervention planning in LMICs can be far from intuitive.

An extensive review of the literature found only one paper that attempted to model any child health data split by wealth quintile. Amouzou et al used the LiST to model child mortality data for richest and poorest quintiles in Bangladesh and found this to be within a 95% confidence interval of current DHS data [32]. This is an impressive result, suggesting that LiST could play a role in expanding knowledge on wealth–related child health outcomes. The paper however does not go further to investigate policy implications and extensive literature searching found no published attempt to adjust any of the major tools (ie, LiST, MBB or CHOICE) for calculating scaling–up costs by wealth quintiles and thereby explore equity considerations. EQUIST appears to be the only published framework which adequately addresses these considerations, making it an important development for future public health policy.

### Limitations

In absence of information, it was necessary to model much of the data needed to populate EQUIST including data for non–antibiotic costs, as although there are several studies estimating overall cost of global scale–up of health systems [18,33] and some discussing the cost of more specific scale–up of individual countries and/or interventions [34,35], no studies were found which reported data on the differential cost of scale–up across wealth quintiles although the importance of this difference was highlighted by Johns and Torres [18].

Estimates of relative disease proportions split by wealth quintile were the most extensive modelling exercise undertaken and are therefore central to consider when assessing this EQUIST implementation’s robustness. The modelling was based on data from the highly–cited CHERG report on child mortality [11] and the UNICEF SOWC 2009 report [36] and is therefore thought to robustly estimate differential disease proportions. That the model used U5MR instead of GDP to split disease distribution is justifiable as the U5MR for Q1–Q5 in each country was known, so this could be used as a common denominator to determine quintile–specific disease distribution.

Limitations of the data in this study are further explained in the Online Supplementary Document(Online Supplementary Document) (table w1).

### Future research/policy implications

The results of this first implementation of EQUIST provide important conclusions. Firstly, one of the main findings of this study was the lack of good data in this important area. The need for extensive future research to fill gaps should be emphasized, especially into variables such as effectiveness and cost of interventions across population wealth strata. One potential way of doing this would be to further expand the DHS or MICS (Multiple Indicator Cluster Surveys [37]) to collect information on more diverse health indicators, including those related to the EQUIST framework variables. This paper also adds to the calls from others for future intervention scale–ups to be monitored with relation to their differential costs, effectiveness and impacts across equity strata so as to widen the knowledge base [38], a process which is starting to happen through the UNICEF initiative “Monitoring Results for Equity System” (MoRES) [39]. The trends observed here for CCM for pneumonia may be similar or completely different for other pneumonia interventions or other major causes of childhood mortality and so if further research was conducted to populate EQUIST with data for other interventions/diseases, these could be investigated and greater understanding could be developed regarding equitable impact of childhood mortality interventions more broadly. For example vaccines have been shown previously to have a positive impact on equity while also reducing childhood mortality significantly, such as in the case of measles vaccination in Bangladesh [40]. Therefore, as vaccines against pneumonia such as Pneumococcal Conjugate (PC) and *Haemophilus influenzae* (Hib) are rolled out across an increasing number of countries through the GAVI Alliance [41,42], using EQUIST it could be possible to target scale–up in a more informed manner, directing vaccines with increased cost–effectiveness while also promoting equity. Further research/modelling however will be necessary to determine the necessary components of the EQUIST model for analysing these interventions before any policy recommendations can be made.

Another potential facet for future research is the inclusion within EQUIST of other indicators of inequity apart from wealth. Policy makers are likely to find targeting interventions strictly by wealth quintiles difficult, therefore decomposing the components of the EQUIST for other sub–population group measures may be of more use. One potential way to do this is to consider using geographical areas to split populations as significant variances in U5MR are typically seen [12-16] and geographical areas are easier for policy makers to target. Further research/modelling however would have to be undertaken to define these groups and their values for each component variable of the EQUIST. Another potential discriminatory variable which could be explored is gender, as U5MR is known to be higher in boys than girls in most LMICs (apart from India and China where the opposite is true) [43], however there is little known with relation to the other variables of EQUIST such as gender differences in disease distribution within specific wealth quintiles. If these data were to be attained either through survey or modelling, it would be possible to apply EQUIST to gender as well as wealth/geography and further address equity considerations.

One of the most important findings in this first implementation of EQUIST is that the current, “mainstream”, approach never showed the highest cost–effectiveness in studied examples. Therefore for CCM scale–up, the current approach is unjustifiable. If countries are already not delivering interventions maximally cost–effectively, and many are increasing inequity, could an equity–focus lead to improvement in both areas? The CCM cost–effectiveness data generated in this paper suggest that indeed the most cost–effective approach is in actuality scale–up in the poorest, as although poorer quintiles display a decrease in effectiveness and an increase in cost of scale–up, the higher burden of disease and case fatality observed in these strata is great enough to offset this. This potentially lends increased weight to policy makers and academics increasingly calling for exactly this kind of equity–focus in scale–up of interventions [2,22,37,43-46]and can be seen as a major development in the evidence supporting this call. Although this implementation is only the first of EQUIST and therefore needs refinement and improvement of data, it is hoped that eventually this tool could be used at a national and sub–national level to aid policy makers to more efficiently target intervention scale–up so as to both save a maximal number of lives and also impact positively on equity.

This implementation of EQUIST and the conceptual process involved behind thinking about intervention scale–up in this manner also suggests possible means of further enhancing cost–effectiveness, resulting in more lives saved for a given investment. The limiting factor in CCM in poorer quintiles such as Nigeria seems to be the very low effectiveness of the intervention and so it is implied that enhancement of the efficiency or quality of provision will also significantly decrease cost and therefore increase cost–effectiveness [6]. This development should therefore be a focus for future research so that cost concerns do not force resource–limited policy makers to further perpetuate the observed trends of increasing inequity in many countries worldwide [2]. A recent review highlights a number of current limiting factors in the effectiveness of community case management including incomplete compliance with guidelines, inappropriate choice of antibiotics and poor management of treatment failure and co–morbidities [47]. These must be overcome if an equitable approach to scaling–up CCM is to become practicable in some of the world’s poorest countries.

### Conclusion

Child health information split by wealth strata in LMICs is severely lacking. This first implementation of the EQUIST framework has expanded knowledge and delivered important analyses on cost–effectiveness of different strategies in scaling up of community case management to tackle pneumonia in five LMICs, demonstrating EQUIST’s potential future value. It has highlighted the complexity of interactions between equity, cost–effectiveness and their determinants, also reinforcing important suggestions for future policy such as the significant effect on cost–effectiveness of increasing efficiency and quality of interventions in poorer quintiles.
