# Supplementary material for: Optimizing community case management strategies to achieve equitable reduction of childhood pneumonia mortality: An application of Equitable Impact Sensitive Tool (EQUIST) in five low– and middle–income countries
Source: J Glob Health. 2012 Dec;2(2):020402. doi: 10.7189/jogh.02.020402 (PMC3529311; doi:10.7189/jogh.02.020402)
Supplement: Online Supplementary Document [file jogh-02-020402-s001.pdf]

Online Supplementary Document

Waters et al. Optimizing community case management strategies to achieve equitable reduction of childhood pneumonia mortality: an application of Equitable Impact Sensitive Tool (EQUIST) in five low and middle-income countries
Journal of Global Health 2012;2:020402

Table w1. Explanation of the sources of information by equity quintiles for different variables used in the EQUIST framework

A. Cost estimates

Antibiotic costs for CCM were taken from a UNICEF report on pneumonia.[8] To estimate non-antibiotic costs for CCM, modelling was based on the assumption that a perfect health system with an U5MR of close to 0 (taken to be 0.5 to enable graphing) would also have non-antibiotic costs of close to 0 (again taken to be 0.5 for the same reason) as no/very little additional health system expenditure would be necessary to deliver CCM. A graph was constructed of U5MR against cost with one point at (0.5,0.5) and the second at a cost of US$1.46 and a U5MR of 90 (the U5MR for the Pakistan study site taken from UNICEF "State of the World's Children" report 2009[23]). It was assumed that cost would robustly follow a linear model of increase, as the additional costs of intervention delivery by equity strata would not be expected to rise much in the poorest quintiles, so a linear trendline was applied between these two points. The equation of this line was then used to calculate non-antibiotic costs in each quintile for each country by using the U5MR of the quintile.

The case fatality rates (CFR) which were used to adjust quintile costs for number of episodes of pneumonia was modelled by calculating country-specific pneumonia case fatality rates from existing data[11] and then graphing this against the known U5MR for each country from SOWC 2009 and subsequently splitting the data by region, resulting in a graph with a trendline for each region.[37] A linear trendline was applied throughout. Although one of the regional graphs showed a slightly higher r^2 value with an exponential trendline, this was marginal and it was assumed that as the worldwide trendline was robustly linear, this higher r^2 finding was an artefact of poor data rather than reflecting an actual difference. The equation of the trendline was used to calculate the CFR for each quintile in each country from the quintile-specific U5MR.

Limitations
Cost modelling presents a potential issue for the robustness of this implementation of EQUIST overall as very little data were known and assumptions were made (see above).

B. Estimates of current coverage by the three interventions by wealth quintiles in five countries

For Nigeria Egypt, Cambodia and Peru the available DHS data were quintile specific and were therefore used directly. For Bangladesh however only overall data was reported.The distribution between the quintiles was performed according to relative number of pneumonia deaths in each quintile, as it was assumed that the CCM coverage percentages for each quintile would follow the quintile's pneumonia mortality level (i.e. if a quintile had lower pneumonia mortality it was then assumed to have higher CCM coverage level).

Limitations:

For all five countries estimates of U5MR and CCM coverage by quintile were obtained from DHS reports, which although extensive have been highlighted previously as potentially biased, with findings of significant under-reporting of indicators in poorer parts of the population.[36] If this is the case it might mean that this paper's estimates of cost-effectiveness for poorer quintiles are overly high. DHS data is also potentially flawed due to relatively small sample sizes. For example, the coverage data for CCM taken from DHS country reports was based on only 805 cases of suspected pneumonia in Egypt and only 690 in Nigeria.[15, 16]It is suggested that future DHS should endeavour to extend surveys to a greater percentage of the population, thereby decreasing the likelihood of poor or skewed data.

C. Effectiveness Estimates

For CCM, linear, polynomial and exponential trendlines were tested on the graph described in the effectiveness step of the methods section. The trendline with the highest R^2 value (i.e., the one which statistically had the greatest predictive value) was applied. The equation of this line was used to calculate the effectiveness of CCM in each quintile for the exemplar countries depending on their U5MR. This was then revised upwards as described above.

Limitations:
While overall effectiveness data for each intervention was known from Theodoratou et al's reviews[11, 12], they were again completely unreported split by wealth quintile in any setting and so it was necessary to model this using expert opinion[32]. Although this is likely to give a valid estimate, significantly more data is needed in the area to confirm this.

D. Disease Proportion Estimates

This was the most complex modelling process undertaken in this study. Firstly, the distributions of under-5 mortality causes by country were established. These data were taken from the most recent CHERG report[11]. The U5MR for each country from the CHERG report was added using data from the UNICEF SOWC 2009 report[22]. Next for each country, the relative proportion of mortality from each cause of death in the CHERG report was calculated by dividing the number of deaths from each cause of death by the total under-5 deaths for each country.

Using the WHO regional groupings from the CHERG report[13], regional graphs were made of relative disease proportion by U5MR for 9 causes of death: AIDS, diarrhoea, malaria, pneumonia, preterm birth complications, birth asphyxia, neonatal sepsis, congenital abnormalities and injury. This is a smaller number than the number of causes listed in the CHERG report but it was opted to move the categories 'Tetanus', 'Measles', 'Meningitis', 'Pertussis', 'Other Infections' and Other Non-Communicable Diseases' into a broad category of 'Other' for the sake of clearer analysis and model stability.

Trendlines were then fitted to each of these graphs. Exponential, linear and logarithmic trendlines were tested on each disease graph and the one with the greatest r^2 value was applied. For birth asphyxia it was found that a 2nd order polynomial trendline exhibited a significantly higher r^2 value than the other models in the regions of Eastern Mediterranean and Southest Asia and so it was decided to apply this to all regions apart from Africa which exhibited a higher r^2 value with a logarithmic trendline. This difference is thought to be potentially due to the absence of a secondary healthcare effect in Africa.

The next step was to use the equations of the trendlines to model relative disease proportions by quintile for this study's five exemplar countries. Individual disease proportions by wealth

quintile were modelled by applying the trendline equations (see above) for each exemplar country's WHO region to the quintile-specific U5MR data described above. The relative proportion of deaths from causes categorised as 'other' for each quintile was calculated by taking the sum of the percentages calculated for the 9 specific causes and subtracting this from 100 (i.e. 100% of mortality). U5MR data were not available for Niue in the Western Pacific region and so this country was removed from the model.

These country specific disease proportion estimates for each of the 5 different wealth quintiles were tabulated and graphed and then applied to the total number of under 5 deaths per year (which was calculated by applying the U5MR for each country to the number of live births for each country, both of which were taken from UNICEF SOWC 2009[23]) to calculate the number of under 5 deaths from each disease in each country in this study's target year, split by wealth quintile.

E. Final Model

The number of deaths that would be saved through each of the approaches to scaling-up an intervention was calculated by multiplying the under 5 pneumonia deaths in 2008 for the particular quintile (as calculated above) by the percentage effectiveness of the intervention estimated in that quintile. This was then brought together with the estimated cost for scaling-up by 10% in each approach to give a measure of cost-effectiveness (cost per life saved (in US$)): the final outcome of the EQUIST.

To model the "mainstream" approach, it was assumed that coverage scale-up would continue to follow current quintile-specific relative distribution and so used the model to split the 10% increase according to current relative distribution between quintiles. Which quintile the next wealthiest 10% scale-up would be in was established from coverage estimates. Calculating the cost of scaling-up by 10% for each approach was done by multiplying cost per quintile scale-up data for the necessary quintile by 0.5 to show how much it would cost to scale-up by 10% (1/2 a quintile). If scaling-up an intervention to the next uncovered 10%, the middle uncovered 10% or through the "mainstream approach" would involve scaling-up parts of more than one quintile then two individual cost calculations were completed for the fraction of the quintiles that would be covered and then the sum of these was calculated.

Note: this supplementary table is provided with the agreement of SSC4 course organiser Simon Riley so as to ensure full understanding and replicability of the methods and results described above.

References relate to the references in the main text of the article.

Table w2. WHO region-specific disease proportion estimates and models for disease proportion against U5MR

1. Africa

		Relative Disease Proportions								
**Country**	**U5MR**	**AIDS**	**Diarrhoea**	**Malaria**	**Pneumonia**	**Preterm birth complications**	**Birth asphyxia**	**Neonatal sepsis**	**Congenital abnormalities**	**Injury**
Mauritius	15.00	0.69	1.73	0.00	7.27	22.84	14.53	5.19	21.80	4.15
Cape Verde	32.00	0.00	8.19	0.29	14.33	24.27	11.40	4.68	9.36	3.22
Algeria	37.00	0.13	12.84	0.00	19.42	21.93	12.67	5.66	7.55	2.32
Botswana	40.00	0.00	6.98	1.04	12.22	22.72	10.57	4.07	10.29	4.97
South Africa	59.00	45.69	8.67	0.07	8.61	12.37	7.05	2.37	3.28	1.90
Comoros	66.00	0.00	20.10	0.00	21.94	16.18	9.50	5.67	2.81	1.89
Namibia	68.00	18.19	6.31	5.11	13.77	18.10	8.37	3.88	5.65	1.61
Eritrea	70.00	4.39	21.37	0.28	19.31	10.72	6.68	4.03	3.28	4.66
Lesotho	84.00	16.95	9.88	0.00	12.81	16.12	13.12	10.40	4.42	2.08
Zimbabwe	90.00	21.19	9.25	3.43	13.29	11.51	8.22	4.34	3.47	1.81
Gabon	91.00	9.71	5.94	29.32	10.86	15.05	8.22	4.23	4.29	1.06
Swaziland	91.00	48.97	8.35	0.03	11.73	8.46	4.52	2.54	2.89	0.97
Sao Tome and Principe	99.00	0.00	14.60	0.81	25.76	12.37	8.11	5.27	3.85	3.04
Togo	100.00	5.76	11.63	25.67	14.76	11.29	8.61	4.75	3.36	1.66
The Gambia	109.00	1.35	13.90	23.19	16.06	11.33	7.79	4.66	2.74	2.84
Malawi	111.00	13.65	10.95	16.64	13.09	10.03	8.09	5.89	2.82	2.45
Madagascar	112.00	0.11	22.46	3.51	21.28	11.34	8.92	6.54	2.33	2.02
Senegal	114.00	1.45	14.80	18.73	17.85	10.31	7.70	4.89	2.97	1.73
Ghana	115.00	2.63	9.49	26.25	10.44	12.11	11.34	9.00	3.71	1.98
Tanzania	116.00	8.51	11.55	16.43	14.29	9.75	9.74	7.51	3.09	3.39
Ethiopia	119.00	2.82	22.82	6.78	15.21	8.85	11.29	8.92	2.07	2.62

Mauritania	119.00	0.54	15.68	13.33	19.55	12.68	9.47	6.39	3.05	2.50
Kenya	121.00	4.69	20.54	10.94	16.09	7.63	7.72	6.08	2.24	3.46
Benin	123.00	1.35	13.02	23.34	19.40	10.14	6.29	2.45	2.98	2.26
Congo (Brazzaville)	125.00	4.68	14.30	23.85	16.02	11.11	6.44	3.74	3.21	1.53
Cote d’Ivoire	127.00	4.10	13.17	21.10	16.70	12.04	8.81	5.80	3.04	1.32
Uganda	130.00	5.02	16.00	22.40	13.55	7.23	6.74	5.11	1.98	3.55
Liberia	133.00	2.79	17.18	15.56	16.97	10.15	8.15	6.09	2.25	1.44
Cameroon	148.00	4.88	16.19	19.05	18.01	8.41	6.46	3.92	2.46	1.90
Guinea	150.00	1.87	13.85	23.62	17.02	7.96	8.12	6.32	2.07	1.40
Angola	158.00	1.78	25.04	8.41	20.01	6.31	5.76	4.61	1.78	2.96
Democratic Republic of the Congo	161.00	1.13	18.55	17.02	20.35	9.88	6.51	4.24	2.72	1.94
Mozambique	168.00	14.04	11.87	12.49	17.61	10.02	8.52	5.59	2.71	1.83
Zambia	170.00	11.95	14.61	15.24	14.74	7.42	7.12	5.84	2.17	3.00
Central African Republic	172.00	6.78	17.29	14.32	20.26	8.61	7.18	4.29	2.36	1.19
Niger	176.00	0.48	20.32	17.95	21.76	7.06	5.64	2.53	1.21	1.71
Burundi	180.00	2.44	23.60	9.25	16.65	6.86	7.49	6.09	1.87	3.87
Rwanda	181.00	0.68	22.57	5.91	15.21	9.08	10.33	8.42	2.56	4.12
Nigeria	189.00	2.74	18.70	20.19	16.46	7.82	7.93	5.78	2.06	1.47
Burkina Faso	191.00	0.85	18.85	20.39	20.82	6.48	5.02	2.66	1.67	2.58
Mali	196.00	1.11	19.18	20.83	18.73	8.38	6.81	3.93	2.07	2.39

Guinea-Bissau	198.00	1.90	19.08	17.65	18.01	7.74	6.12	5.07	1.66	2.05
Equatorial Guinea	206.00	3.08	9.37	27.86	11.79	10.24	7.39	4.40	3.39	1.35
Chad	209.00	2.89	21.85	18.59	19.28	5.78	6.43	2.92	1.30	1.47
Sierra Leone	262.00	1.50	20.87	12.94	19.79	7.66	5.76	3.66	1.26	2.51

Models for disease proportion against U5MR

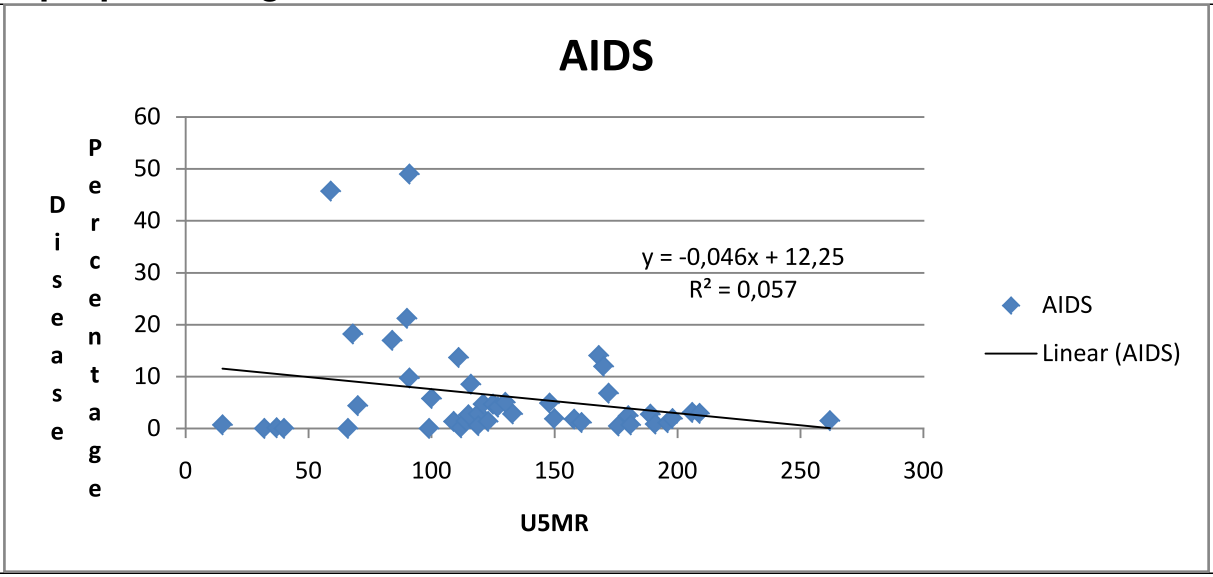

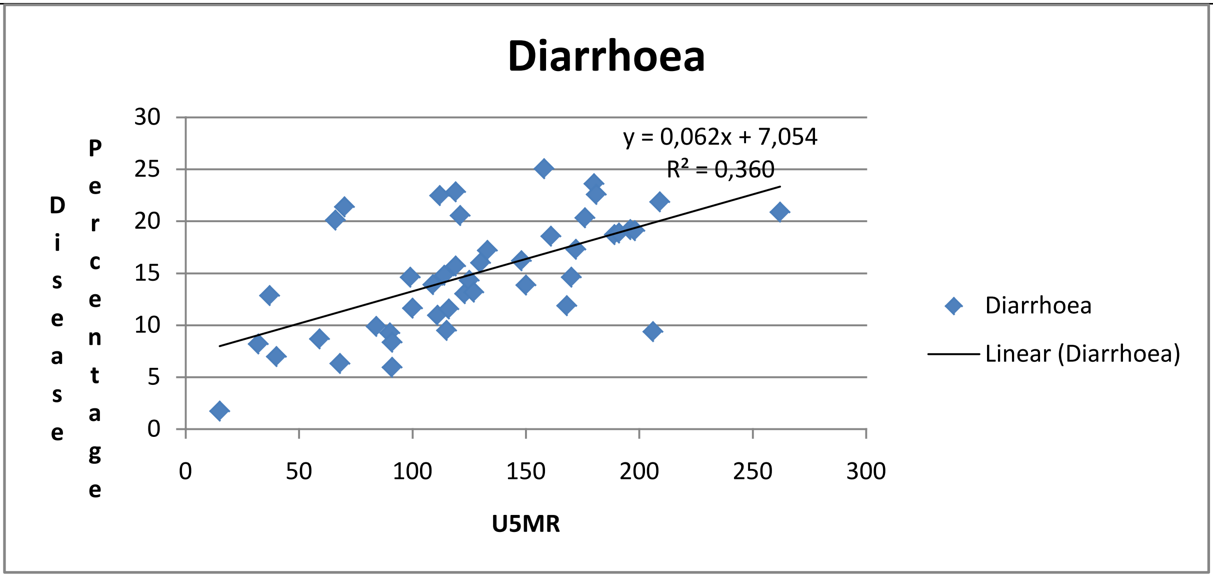
Diarrhoea
y = 0,062x + 7,054
R² = 0,360
Percentage
Disease
U5MR
Diarrhoea
Linear (Diarrhoea)

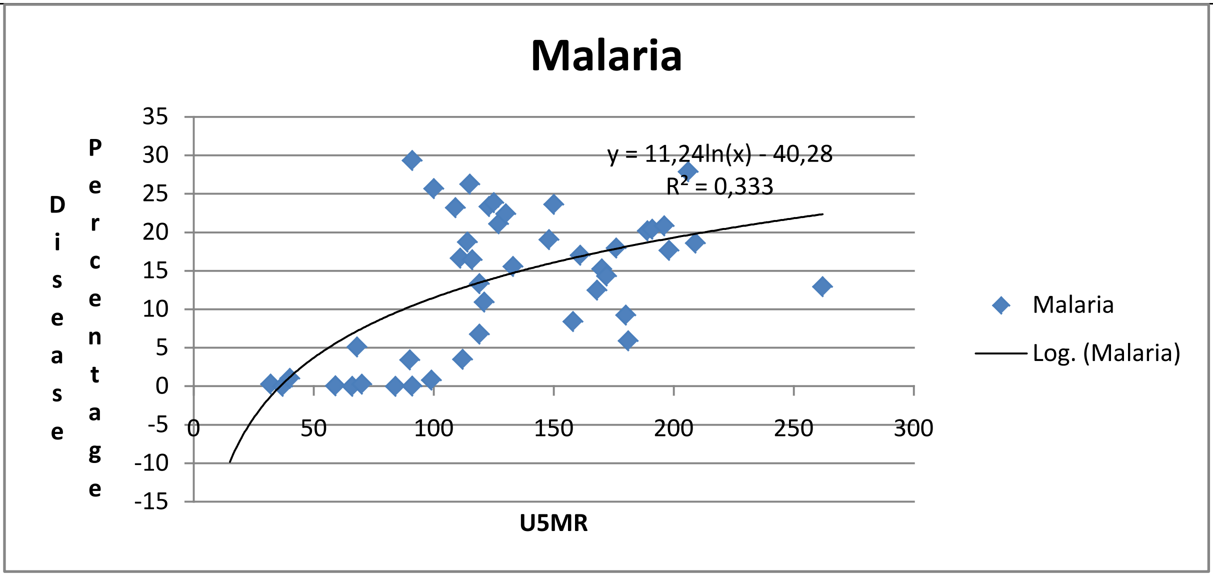
Malaria
y = 11,24ln(x) - 40,28
R² = 0,333
Percentage
Disease
U5MR
Malaria
Log. (Malaria)

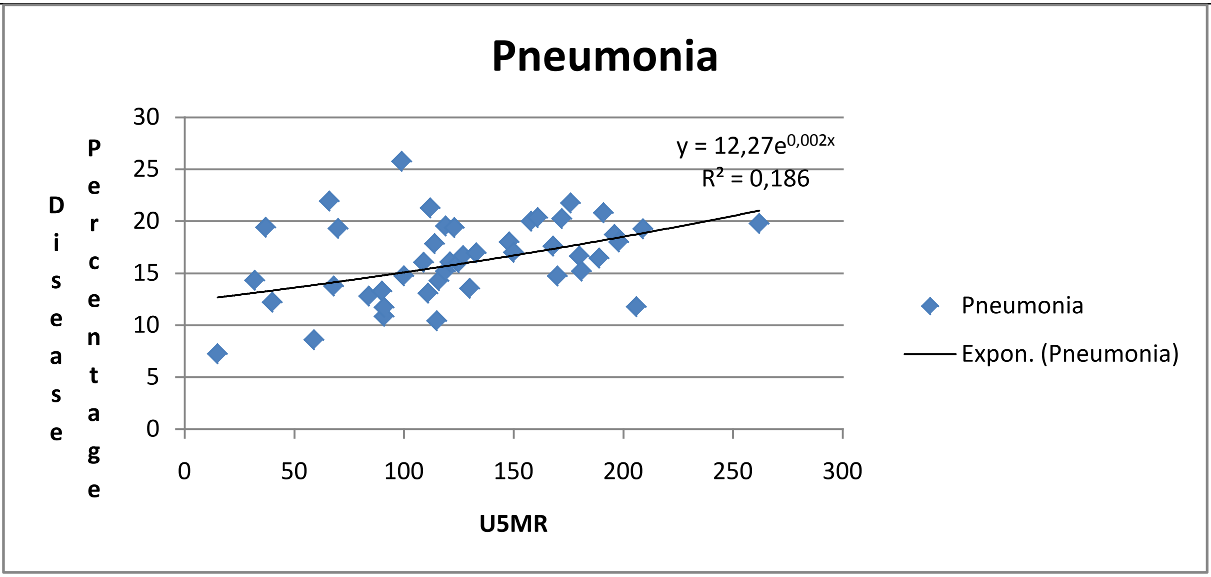
Pneumonia
Disease Percentage
30
25
20
15
10
5
0
0
50
100
150
200
250
300
U5MR
y = 12,27e0,002x
R² = 0,186
Pneumonia
Expon. (Pneumonia)

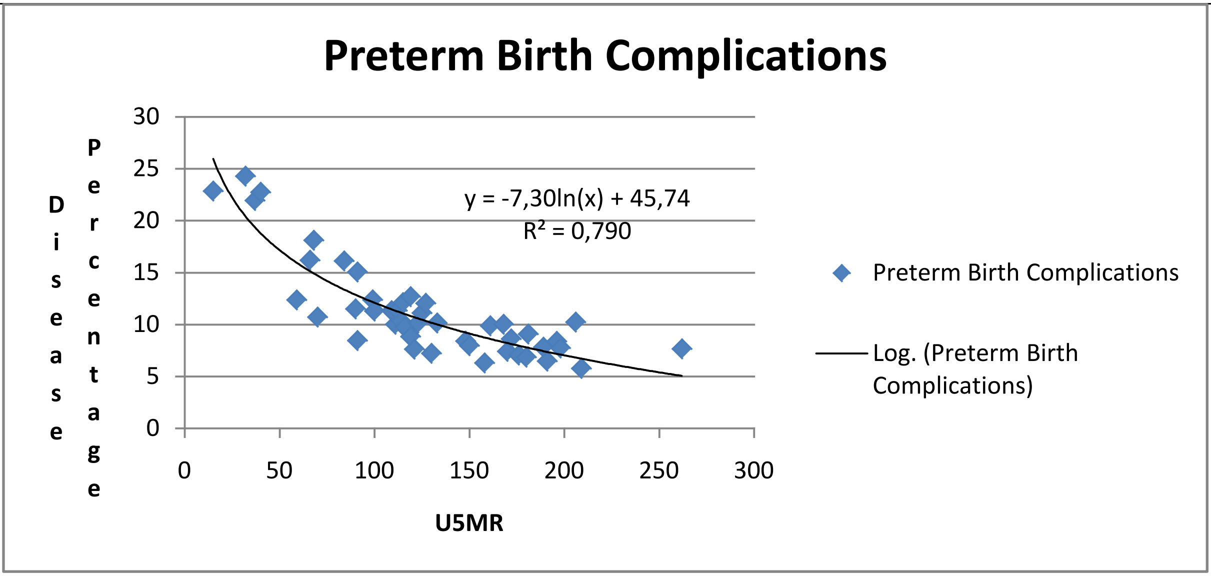
Preterm Birth Complications
Disease Percentage
30
25
20
15
10
5
0
0
50
100
150
200
250
300
U5MR
y = -7,30ln(x) + 45,74
R² = 0,790
Preterm Birth Complications
Log. (Preterm Birth Complications)

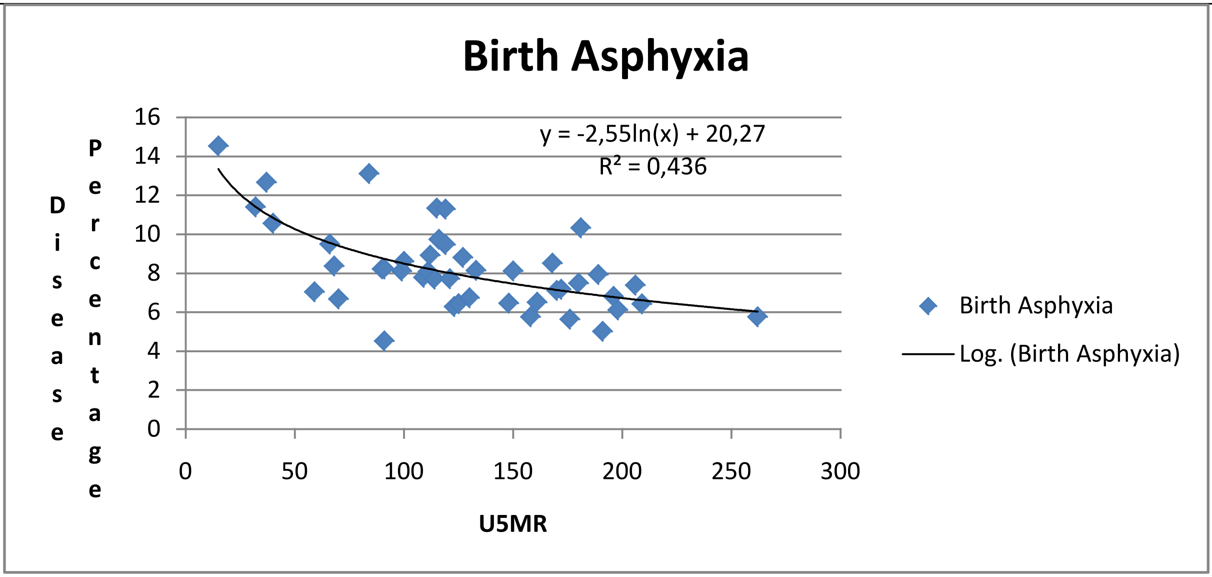

Birth Asphyxia
y = -2,55ln(x) + 20,27
R² = 0,436
Disease Percentage
16
14
12
10
8
6
4
2
0
0 50 100 150 200 250 300
U5MR
Birth Asphyxia
Log. (Birth Asphyxia)

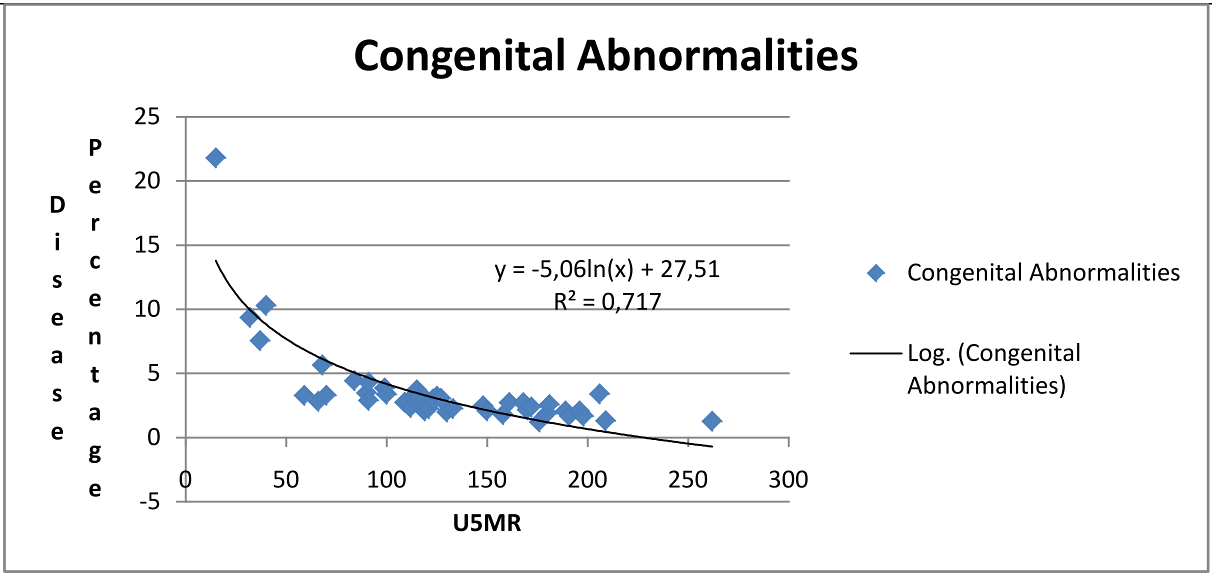

Congenital Abnormalities
y = -5,06ln(x) + 27,51
R² = 0,717
Disease Percentage
25
20
15
10
5
0
-5
0 50 100 150 200 250 300
U5MR
Congenital Abnormalities
Log. (Congenital Abnormalities)

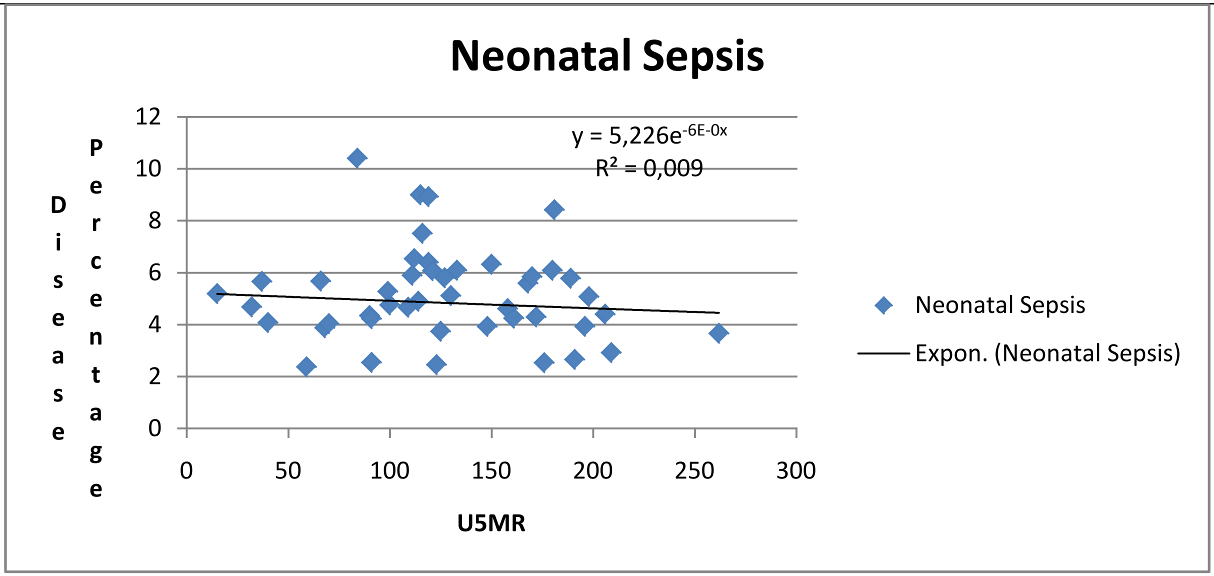
Neonatal Sepsis
y = 5,226e-6E-0x
R² = 0,009
Disease Percentage
U5MR
Neonatal Sepsis
Expon. (Neonatal Sepsis)

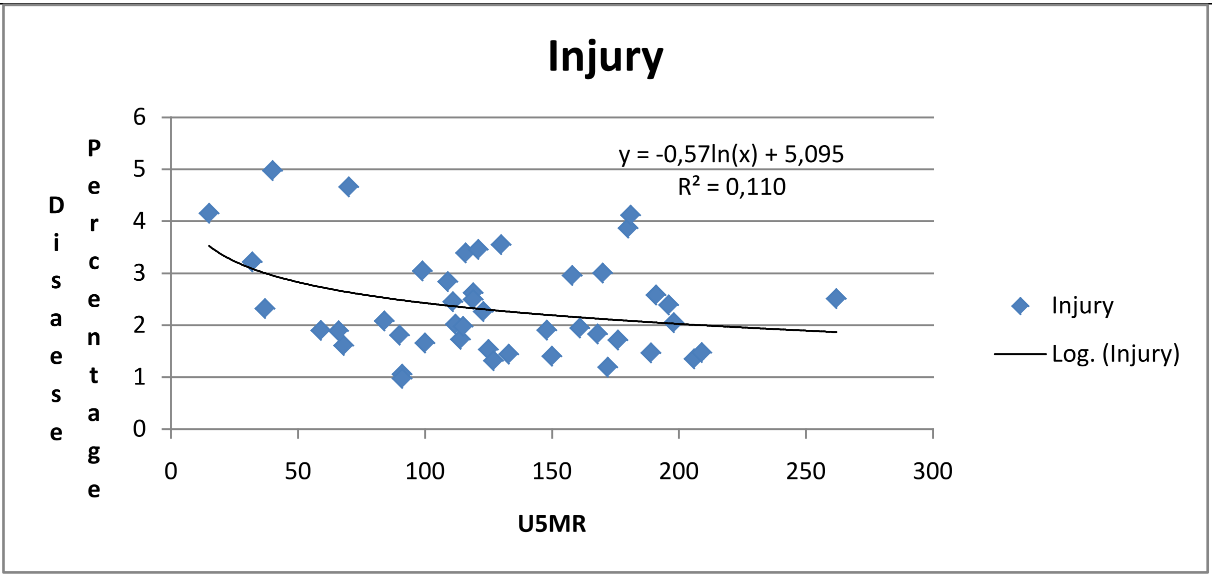

2. Eastern Mediterranean

		Relative Disease Proportions								
**Country**	**U5MR**	**Aids**	**Diarrhoea**	**Malaria**	**Pneumonia**	**Preterm birth complications**	**Birth asphyxia**	**Neonatal sepsis**	**Congenital abnormalities**	**Injury**
United Arab Emirates	8.00	0.00	0.87	0.00	4.99	31.45	5.64	1.08	29.07	7.38
Bahrain	10.00	0.00	0.58	0.00	1.17	23.39	6.43	1.17	42.69	9.94
Kuwait	11.00	0.00	0.54	0.00	3.77	28.19	3.23	1.62	47.58	4.49
Oman	12.00	0.00	1.86	0.00	6.72	32.19	6.29	1.14	25.46	7.01
Qatar	15.00	0.00	1.64	0.00	5.74	24.59	4.92	0.82	26.23	11.48
Syrian Arab Republic	17.00	0.00	4.66	0.00	10.94	26.22	5.44	0.94	21.56	7.26
Libya	18.00	0.00	3.66	0.00	8.79	29.86	6.04	1.11	21.89	7.35

Tunisia	21.00	0.00	4.79	0.00	10.40	30.43	5.87	1.23	20.46	5.90
Jordan	24.00	0.00	3.88	0.00	10.72	35.24	6.19	1.56	19.48	4.56
Saudi Arabia	25.00	0.00	4.79	0.00	9.84	31.47	5.89	1.29	18.88	8.48
Lebanon	29.00	0.70	2.44	0.00	7.67	30.23	5.93	1.05	23.49	7.56
Iran (Islamic Republic of)	33.00	0.53	9.83	0.00	16.05	26.55	8.79	3.86	14.09	4.06
Morocco	34.00	0.09	12.36	0.00	17.22	20.57	14.58	8.10	9.99	2.65
Egypt	36.00	0.07	4.79	0.00	10.60	33.04	5.62	1.33	18.18	4.55
Iraq	44.00	0.00	11.55	0.00	20.33	22.61	11.70	4.63	8.21	5.08
Yemen	73.00	0.00	20.20	0.46	18.04	16.77	11.67	5.28	4.90	2.54
Pakistan	90.00	0.09	15.96	0.12	18.11	16.65	14.54	12.12	6.82	1.72
Sudan	109.00	2.42	10.64	24.89	15.56	18.06	6.92	1.81	3.60	2.77
Djibouti	127.00	6.07	18.65	0.18	18.61	11.24	9.01	6.29	7.23	2.32
Somalia	142.00	0.22	21.83	5.85	18.99	8.12	8.45	5.45	2.84	2.50
Afghanistan	257.00	0.01	28.87	0.01	25.97	4.58	5.20	4.18	1.86	3.90
**Models for disease proportion against U5MR**										

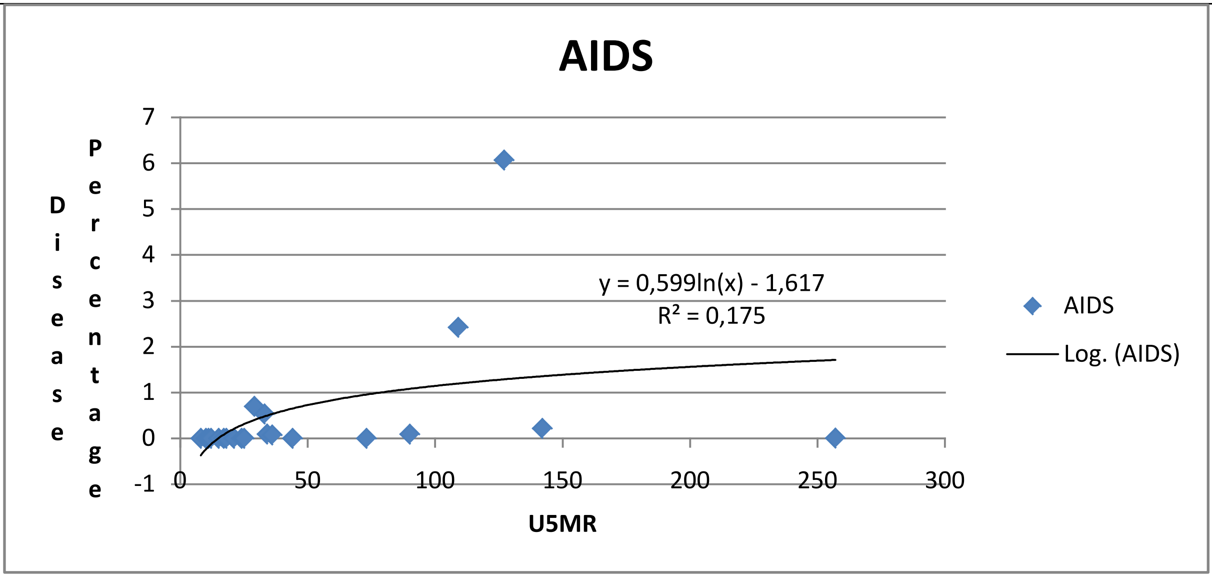
AIDS
Percentage
Disease
7
6
5
4
3
2
1
0
-1
0
50
100
150
200
250
300
U5MR
y = 0,599ln(x) - 1,617
R² = 0,175
AIDS
Log. (AIDS)

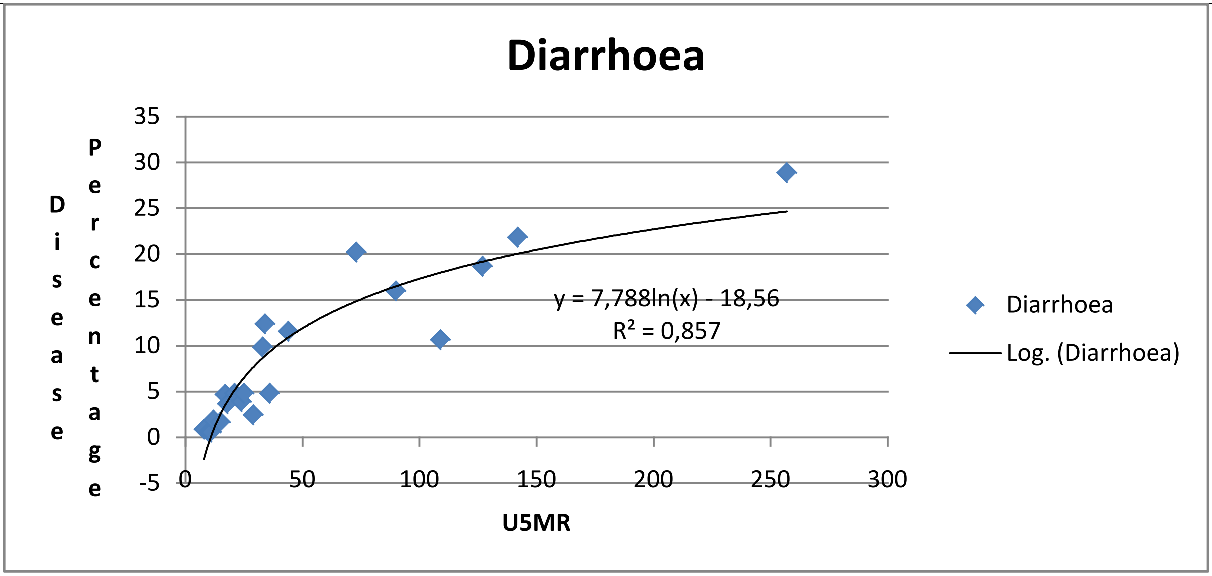
Diarrhoea
Percentage
Disease
35
30
25
20
15
10
5
0
-5
0
50
100
150
200
250
300
U5MR
y = 7,788ln(x) - 18,56
R² = 0,857
Diarrhoea
Log. (Diarrhoea)

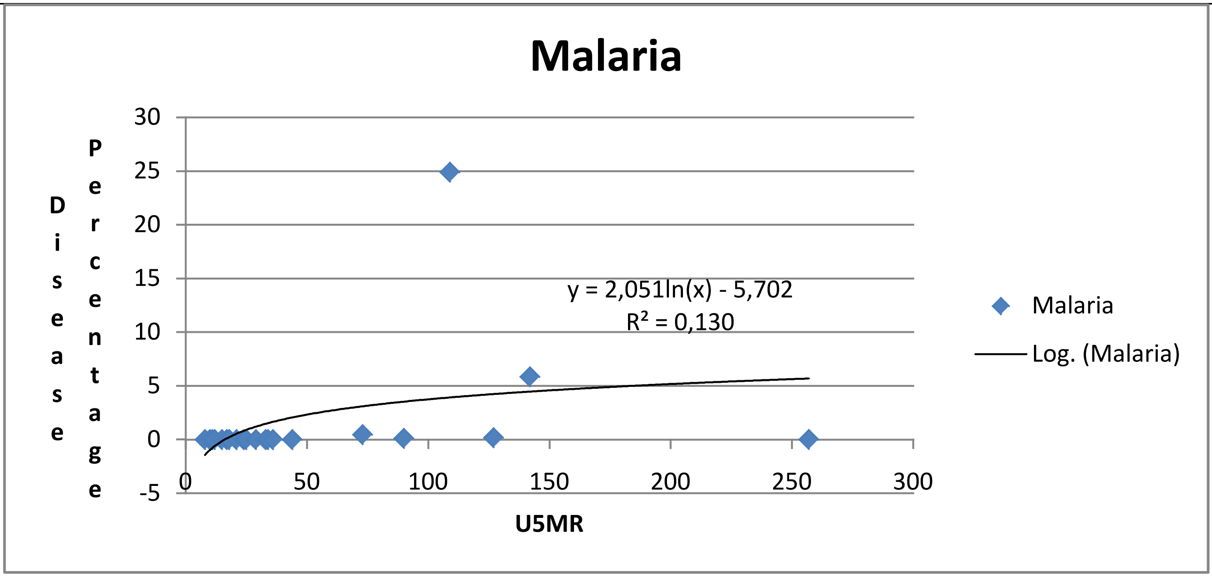
Malaria
Disease Percentage
30
25
20
15
10
5
0
-5
0
50
100
150
200
250
300
U5MR
y = 2,051ln(x) - 5,702
R² = 0,130
Malaria
Log. (Malaria)

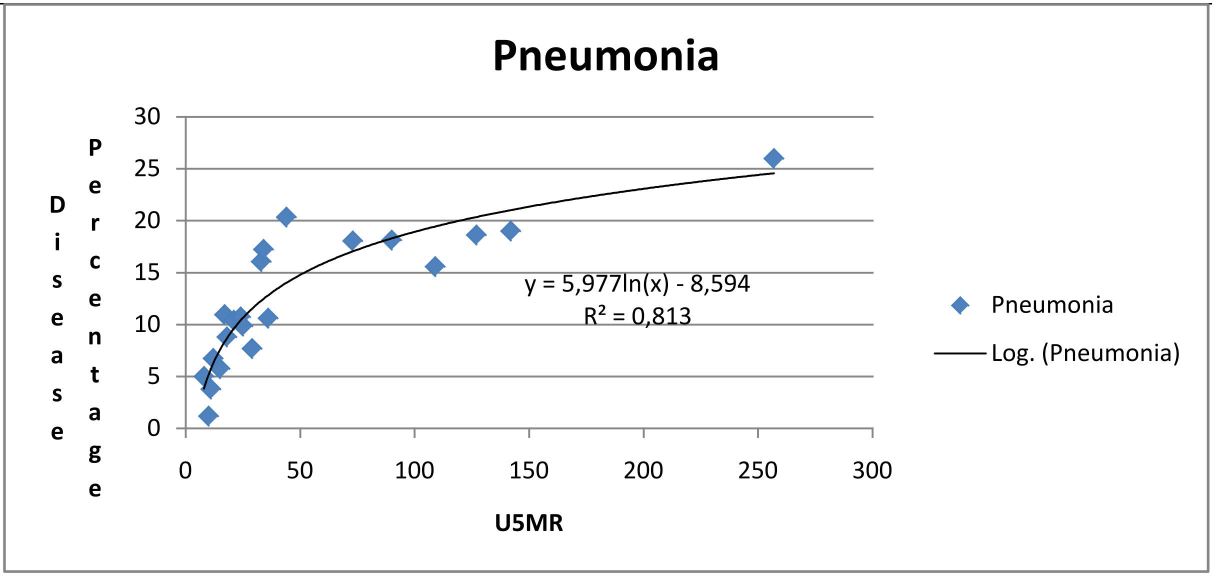
Pneumonia
Disease Percentage
30
25
20
15
10
5
0
0
50
100
150
200
250
300
U5MR
y = 5,977ln(x) - 8,594
R² = 0,813
Pneumonia
Log. (Pneumonia)

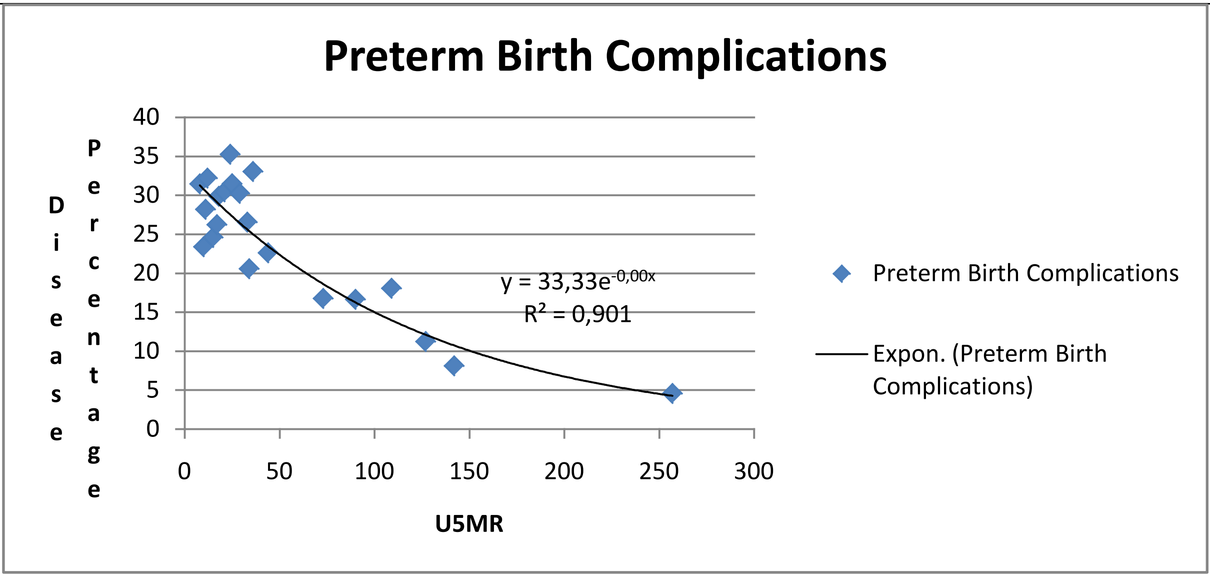
Preterm Birth Complications
Disease
Percentage
U5MR
y = 33,33e$^{-0,00x}$
R^2 = 0,901
Preterm Birth Complications
Expon. (Preterm Birth Complications)

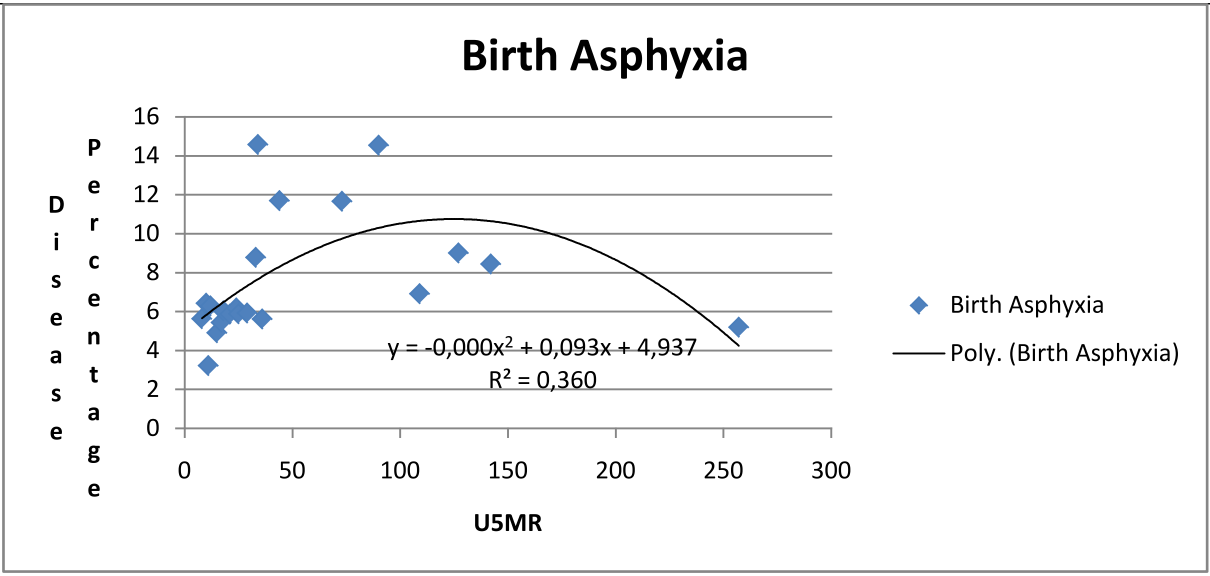
Birth Asphyxia
Disease
Percentage
U5MR
y = -0,000x^2 + 0,093x + 4,937
R^2 = 0,360
Birth Asphyxia
Poly. (Birth Asphyxia)

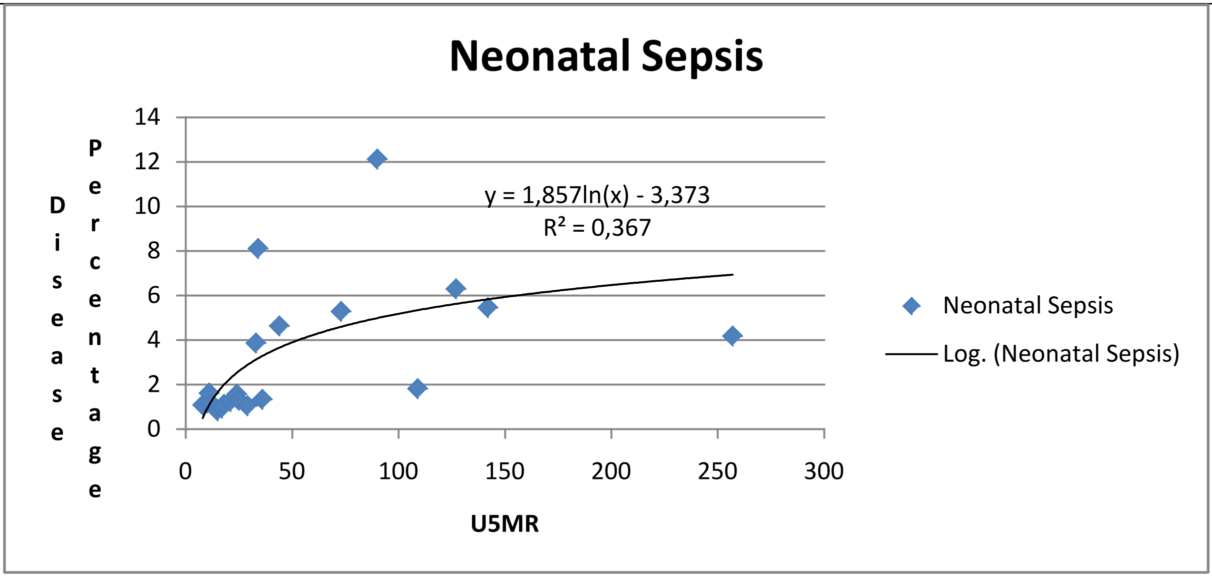
Neonatal Sepsis
Percentage Disease
14
12
10
8
6
4
2
0
0
50
100
150
200
250
300
U5MR
y = 1,857ln(x) - 3,373
R² = 0,367
Neonatal Sepsis
Log. (Neonatal Sepsis)

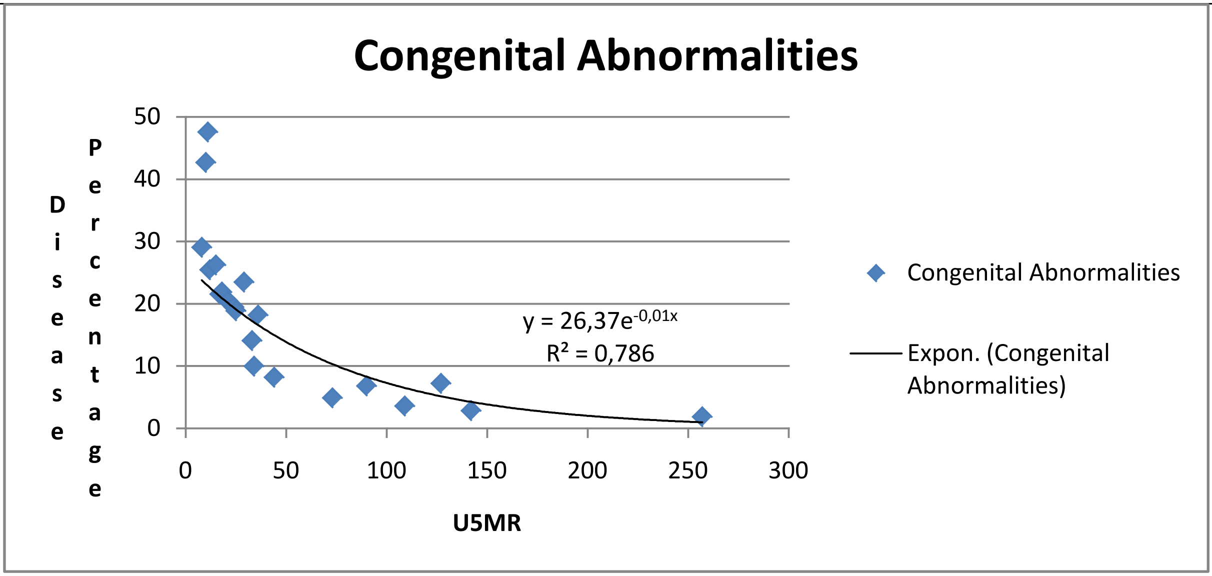
Congenital Abnormalities
Percentage Disease
50
40
30
20
10
0
0
50
100
150
200
250
300
U5MR
y = 26,37e-0,01x
R² = 0,786
Congenital Abnormalities
Expon. (Congenital Abnormalities)

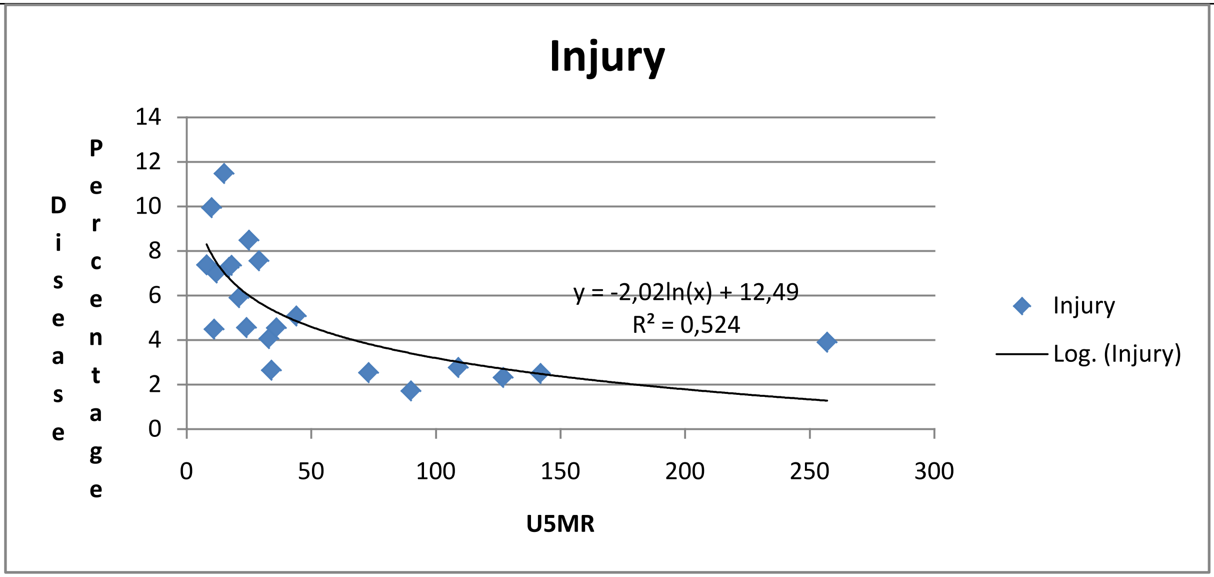
Injury
Disease
Percentage
14
12
10
8
6
4
2
0
0
50
100
150
200
250
300
U5MR
y = -2,02ln(x) + 12,49
R² = 0,524
Injury
Log. (Injury)

3. Western Pacific

		Relative Disease Proportions								
**Country**	**U5MR**	**Aids**	**Diarrhoea**	**Malaria**	**Pneumonia**	**Preterm birth complications**	**Birth asphyxia**	**Neonatal sepsis**	**Congenital abnormalities**	**Injury**
Singapore	3.00	0.00	0.00	0.00	12.61	25.23	0.90	2.70	35.14	5.41
Japan	4.00	0.00	1.27	0.00	6.22	8.87	4.92	1.82	38.67	10.69
Republic of Korea	5.00	0.00	0.21	0.00	3.57	26.15	5.02	3.53	20.96	12.16
Australia	6.00	0.00	0.36	0.00	2.27	22.46	9.31	1.42	23.67	7.75
New Zealand	6.00	0.00	0.28	0.00	4.72	25.83	10.56	3.33	24.72	9.72
Brunei Darussalam	9.00	0.00	1.96	0.00	1.96	13.73	7.84	1.96	35.29	13.73
Malaysia	11.00	3.72	0.99	0.17	6.11	21.77	8.50	1.11	25.89	8.53
Vietnam	15.00	3.27	2.26	0.13	9.98	26.96	10.28	1.76	19.32	4.64
Fiji	18.00	0.64	5.43	0.00	13.10	22.68	8.63	1.28	17.57	7.03
China	22.00	0.32	3.09	0.00	16.85	15.18	16.82	1.93	10.02	10.69
Tonga	23.00	0.00	7.14	0.00	16.07	19.64	8.93	1.79	16.07	7.14
Samoa	27.00	0.00	7.41	0.00	19.44	20.37	8.33	1.85	11.11	5.56
Philippines	28.00	0.04	6.67	0.05	23.85	19.08	6.89	1.74	8.41	4.29
Vanuatu	34.00	0.00	6.58	2.19	23.25	19.74	7.46	1.32	8.33	3.95
Federated States of Micronesia	40.00	0.00	4.59	0.00	29.36	17.43	7.34	2.75	5.50	3.67
Mongolia	43.00	0.10	4.52	0.00	28.52	13.75	5.11	1.47	6.58	5.01

Marshall Islands	54.00	0.00	8.51	0.00	23.40	17.02	6.38	2.13	8.51	4.26
Kiribati	63.00	0.00	17.00	0.00	24.00	15.00	8.00	3.00	7.00	4.00
Papua New Guinea	65.00	2.59	5.21	7.29	22.44	11.37	11.40	6.22	3.37	3.03
Lao People's Democratic Republic	70.00	0.10	7.42	0.19	26.79	8.61	10.60	5.51	3.63	2.86
Solomon Islands	70.00	0.00	3.83	6.20	25.73	17.88	6.20	1.64	6.39	2.92
Cambodia	91.00	0.33	7.13	0.78	28.12	10.99	10.00	7.55	2.30	3.44

Models for disease proportion against U5MR

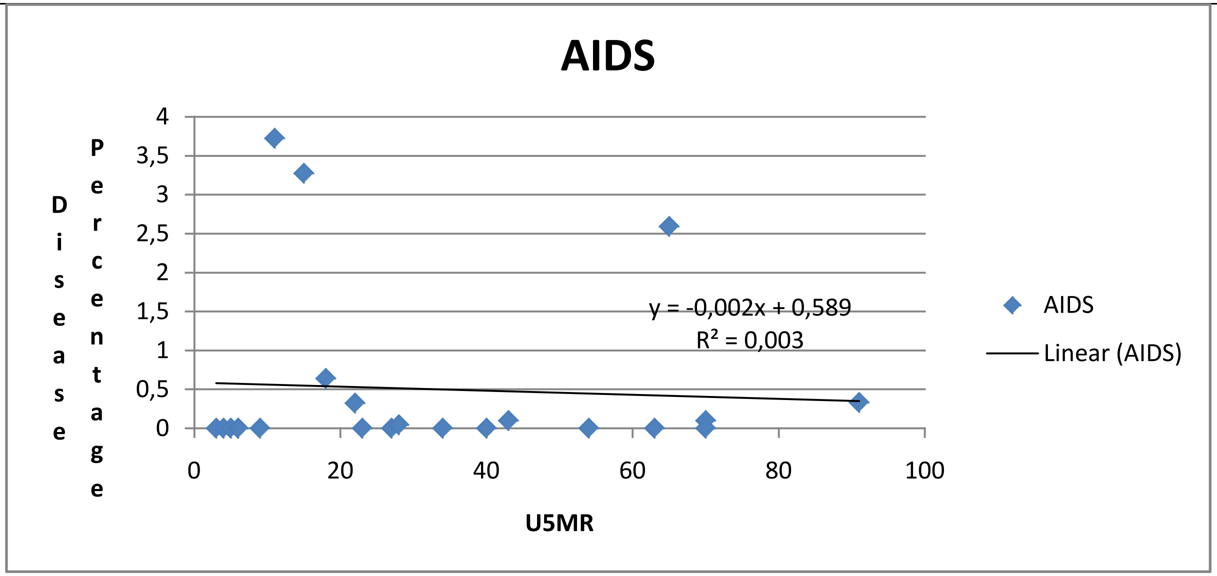
AIDS
Disease
Percentage
4
3,5
3
2,5
2
1,5
1
0,5
0
0
20
40
60
80
100
U5MR
y = -0,002x + 0,589
R² = 0,003
AIDS
Linear (AIDS)

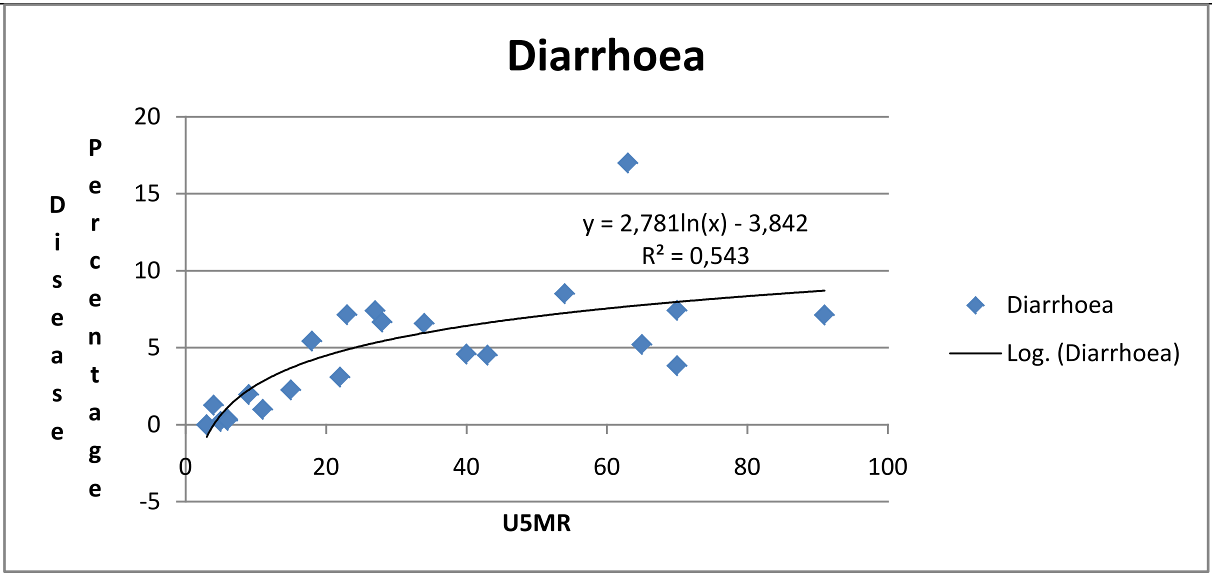
Diarrhoea
20
15
10
5
0
-5
0
20
40
60
80
100
y = 2,781ln(x) - 3,842
R² = 0,543
Diarrhoea
Log. (Diarrhoea)
Disease Percentage
U5MR

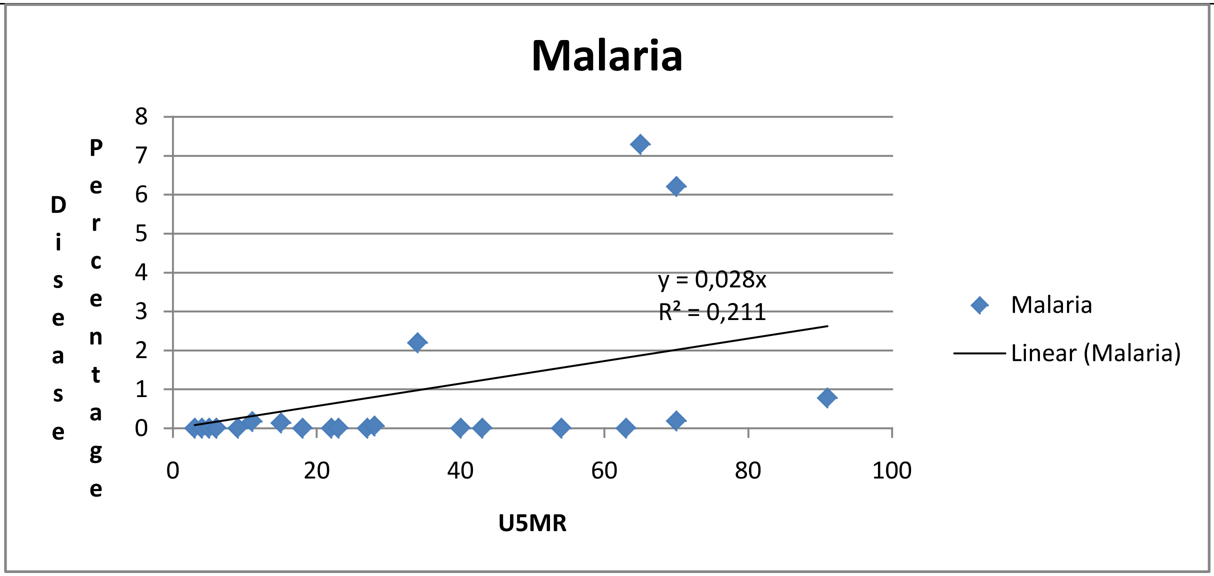

Malaria
Disease Percentage
8
7
6
5
4
3
2
1
0
0
20
40
60
80
100
U5MR
y = 0,028x
R² = 0,211
Malaria
Linear (Malaria)

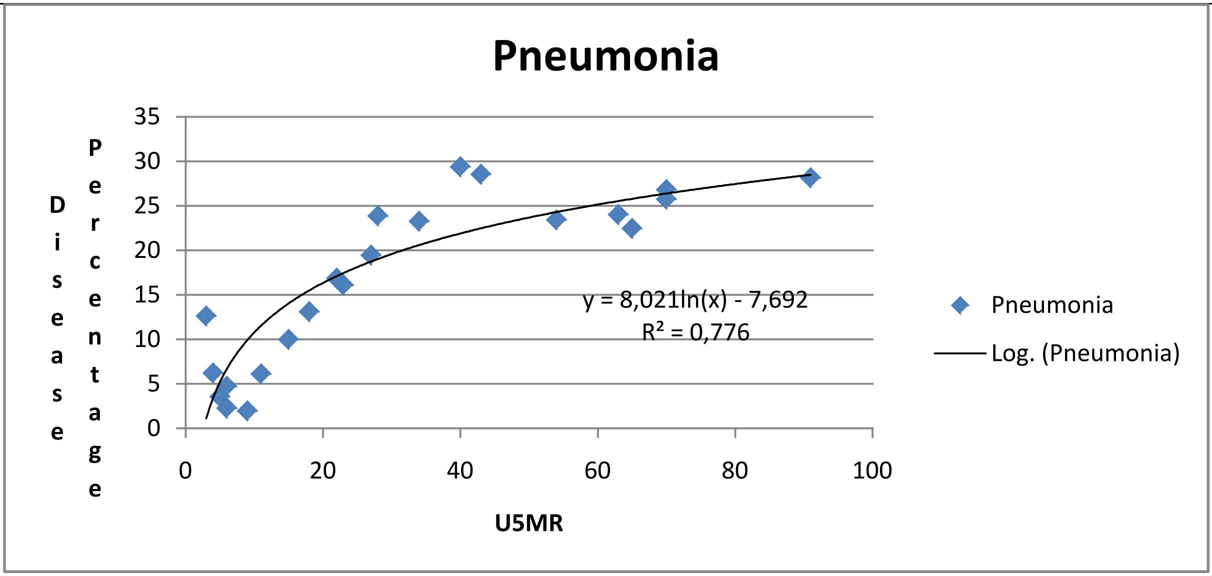
Pneumonia
Disease Percentage
35
30
25
20
15
10
5
0
0
20
40
60
80
100
U5MR
y = 8,021ln(x) - 7,692
R² = 0,776
Pneumonia
Log. (Pneumonia)

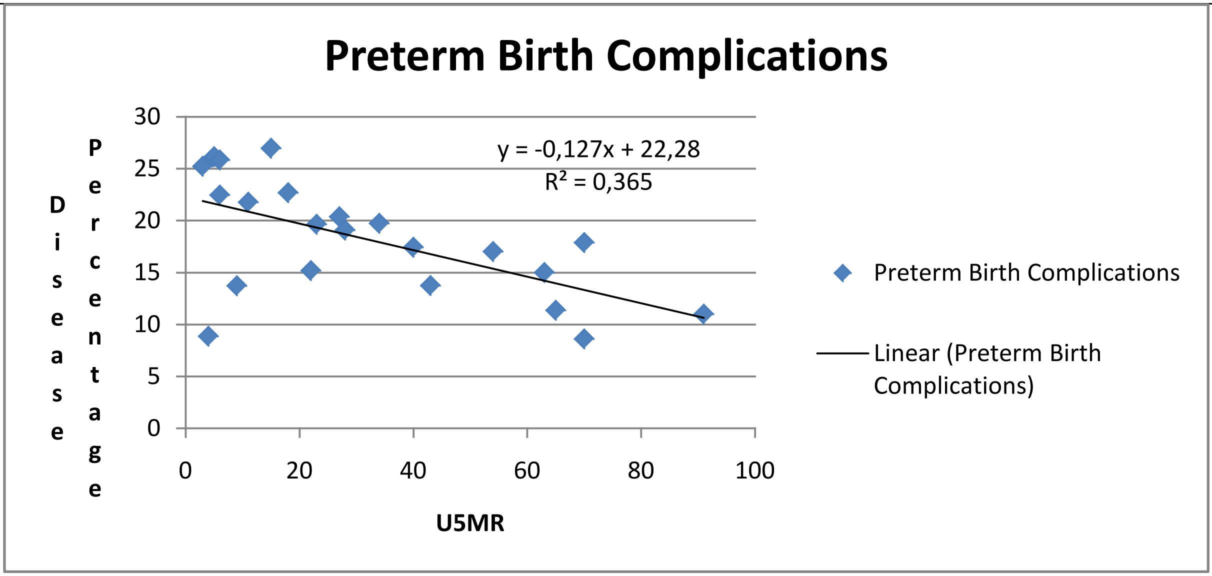
Preterm Birth Complications
Disease
Percentage
U5MR
y = -0,127x + 22,28
R² = 0,365
Preterm Birth Complications
Linear (Preterm Birth Complications)

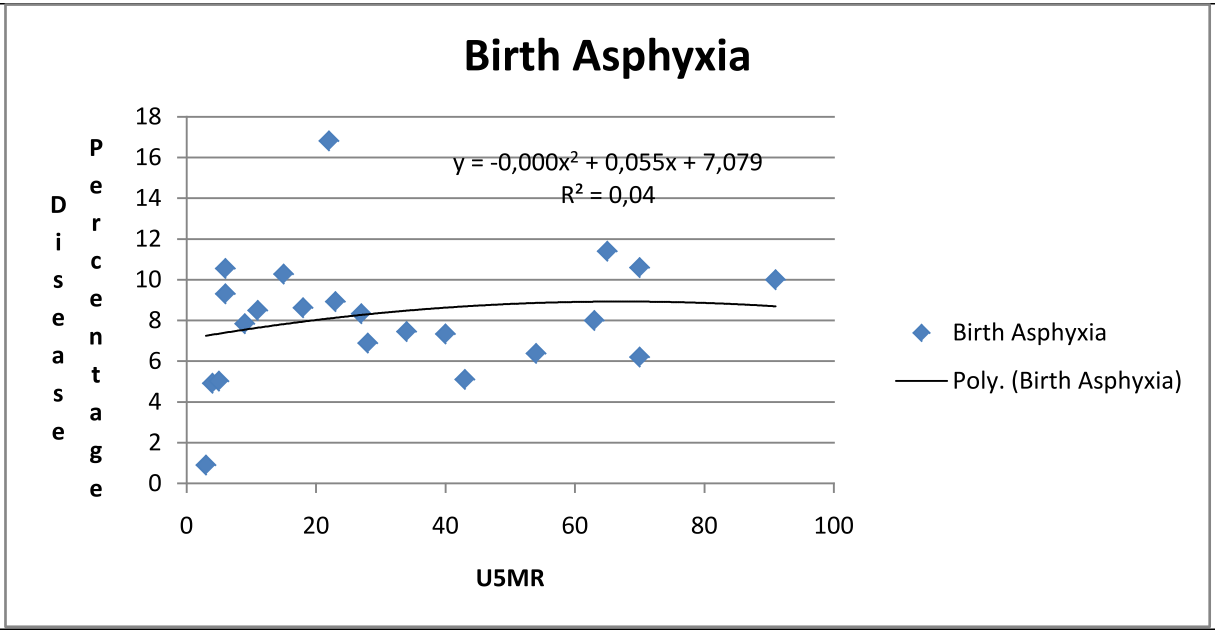

Birth Asphyxia
y = -0,000x² + 0,055x + 7,079
R² = 0,04
Disease Percentage
U5MR
Birth Asphyxia
Poly. (Birth Asphyxia)

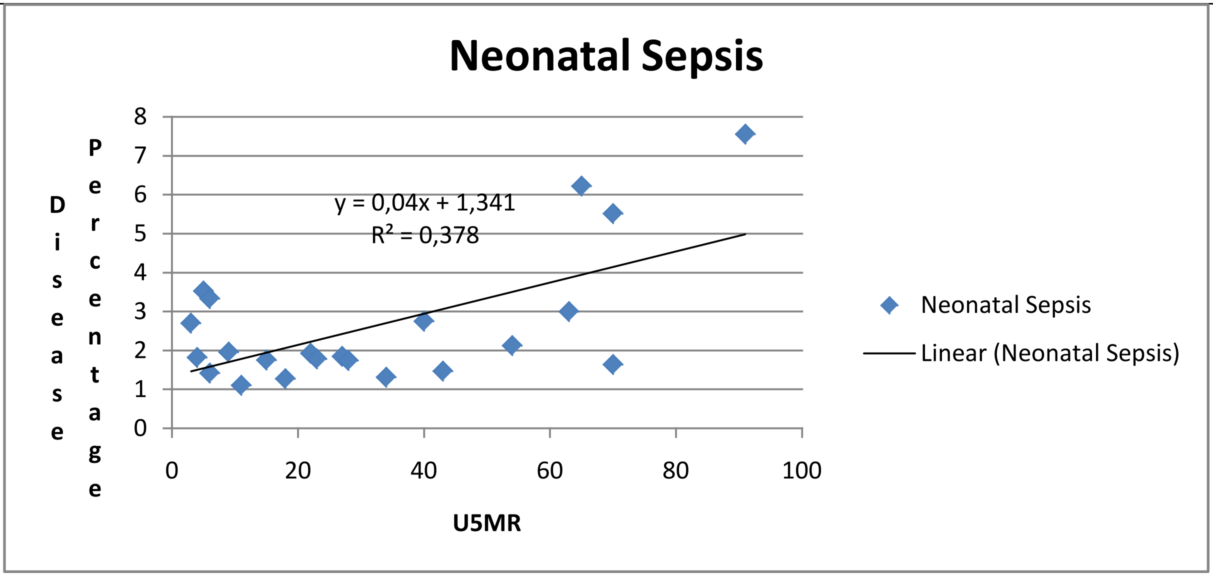
Neonatal Sepsis
y = 0,04x + 1,341
R² = 0,378
Disease Percentage
U5MR
Neonatal Sepsis
Linear (Neonatal Sepsis)

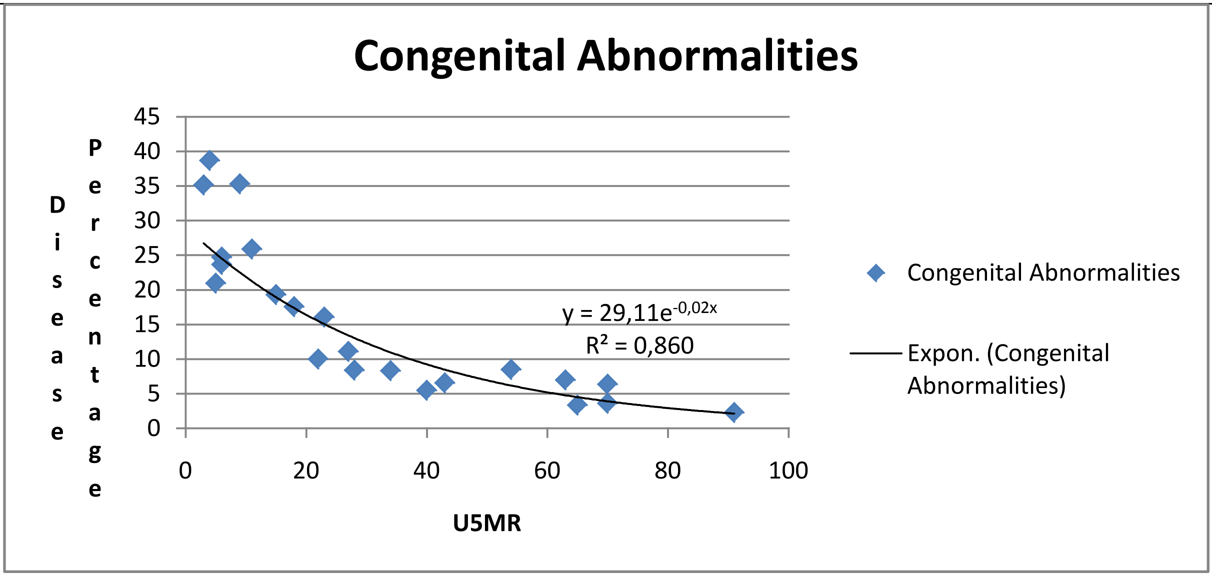
Congenital Abnormalities
Percentage
Disease
45
40
35
30
25
20
15
10
5
0
0
20
40
60
80
100
U5MR
y = 29,11e^-0,02x
R² = 0,860
Congenital Abnormalities
Expon. (Congenital Abnormalities)

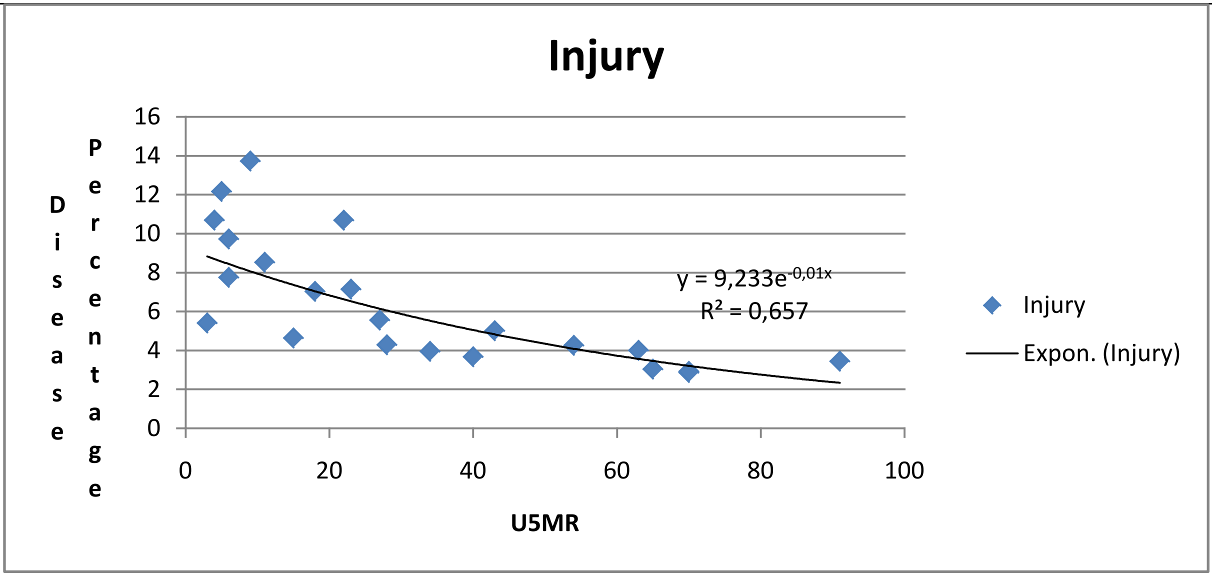

Injury
16
14
12
10
8
6
4
2
0
Disease Percentage
0
20
40
60
80
100
U5MR
y = 9,233e-0,01x
R² = 0,657
Injury
Expon. (Injury)

4. Southeast Asia

		Relative Disease Proportions								
**Country**	**U5MR**	**Aids**	**Diarrhoea**	**Malaria**	**Pneumonia**	**Preterm birth complications**	**Birth asphyxia**	**Neonatal sepsis**	**Congenital abnormalities**	**Injury**
Thailand	7.00	1.95	1.90	0.50	10.46	29.75	11.22	2.35	20.64	4.58
Sri Lanka	21.00	0.10	3.24	0.00	10.37	22.15	8.19	1.47	18.51	16.11
Maldives	30.00	0.00	8.59	0.00	15.95	25.77	11.04	4.91	9.20	4.29
Indonesia	31.00	0.37	15.10	0.80	22.15	19.22	10.12	4.99	5.55	2.05
Nepal	55.00	0.76	14.71	0.02	14.39	16.75	17.90	14.40	3.91	2.19
Democratic People's Republic of Korea	55.00	0.08	12.07	0.00	20.00	21.12	11.67	7.00	6.32	2.45
Bangladesh	61.00	0.03	11.02	1.77	14.20	16.70	17.70	16.11	3.71	2.35
India	72.00	0.47	12.98	0.34	20.31	13.61	10.47	6.63	3.00	2.92
Bhutan	84.00	0.00	14.10	0.00	24.27	13.93	10.76	7.26	3.75	4.09
Timor-Leste	97.00	0.00	13.48	11.44	10.42	12.36	16.03	11.52	3.11	2.29
Burma/Myanmar	103.00	0.54	12.82	1.75	13.19	13.99	10.66	8.74	2.23	22.40

Models for disease proportion against U5MR

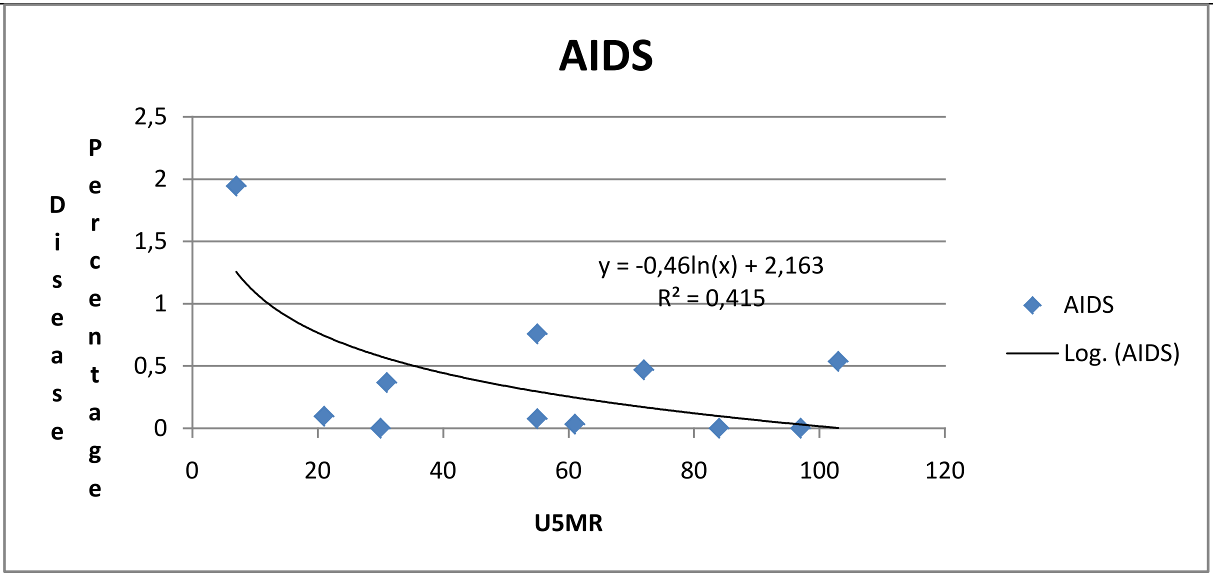
AIDS
Disease Percentage
2,5
2
1,5
1
0,5
0
0
20
40
60
80
100
120
U5MR
y = -0,46ln(x) + 2,163
R² = 0,415
AIDS
Log. (AIDS)

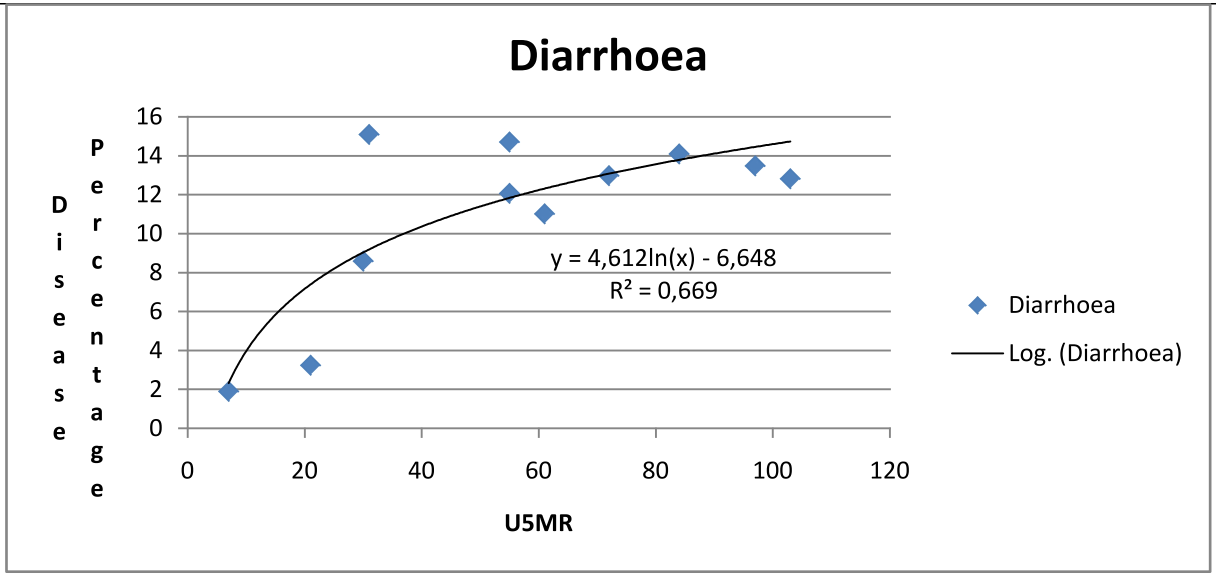
Diarrhoea
Disease Percentage
U5MR
y = 4,612ln(x) - 6,648
R² = 0,669
Diarrhoea
Log. (Diarrhoea)

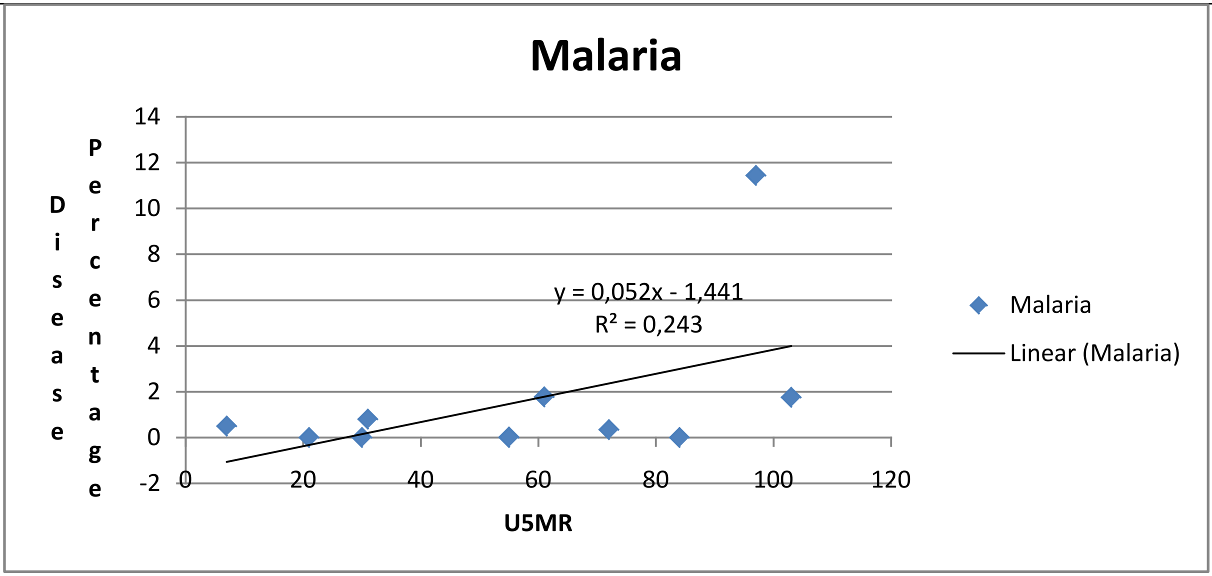
Malaria
Disease Percentage
14
12
10
8
6
4
2
0
-2
0
20
40
60
80
100
120
U5MR
y = 0,052x - 1,441
R² = 0,243
Malaria
Linear (Malaria)

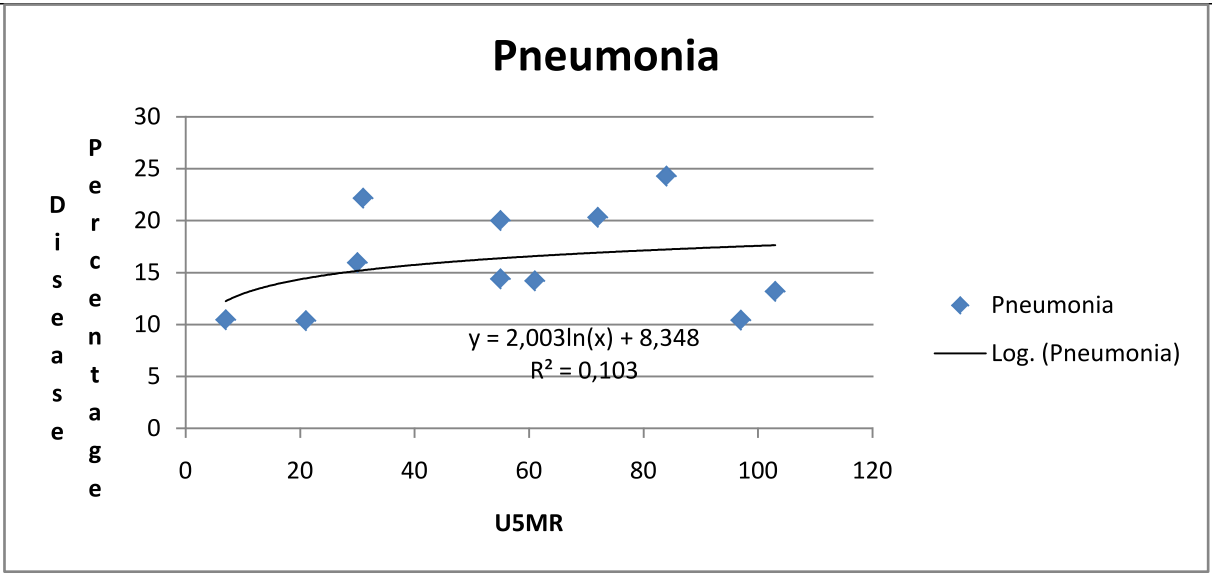
Pneumonia
Disease Percentage
30
25
20
15
10
5
0
0
20
40
60
80
100
120
U5MR
y = 2,003ln(x) + 8,348
R² = 0,103
Pneumonia
Log. (Pneumonia)

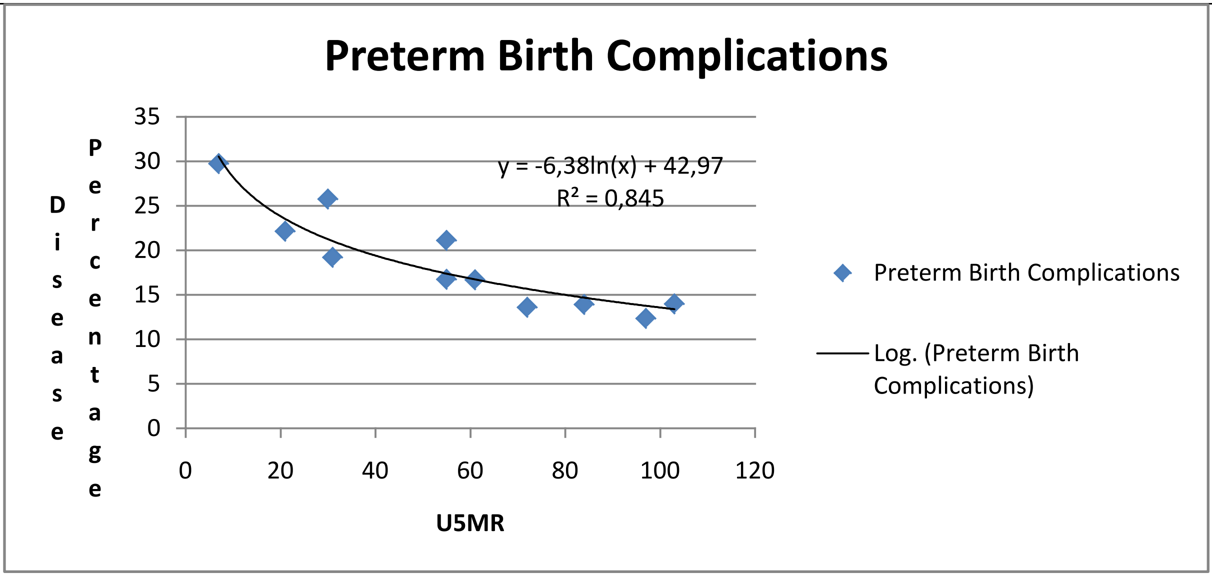
Preterm Birth Complications
Disease Percentage
U5MR
y = -6,38ln(x) + 42,97
R² = 0,845
Preterm Birth Complications
Log. (Preterm Birth Complications)
0
5
10
15
20
25
30
35
0
20
40
60
80
100
120

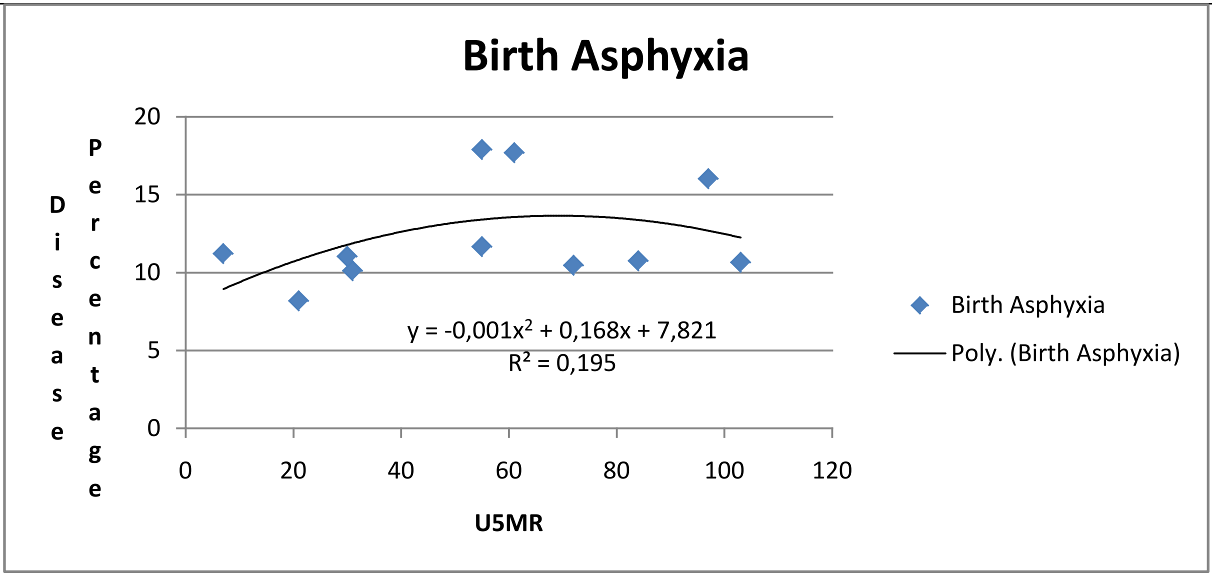

Birth Asphyxia
Disease Percentage
20
15
10
5
0
0
20
40
60
80
100
120
U5MR
y = -0,001x² + 0,168x + 7,821
R² = 0,195
Birth Asphyxia
Poly. (Birth Asphyxia)

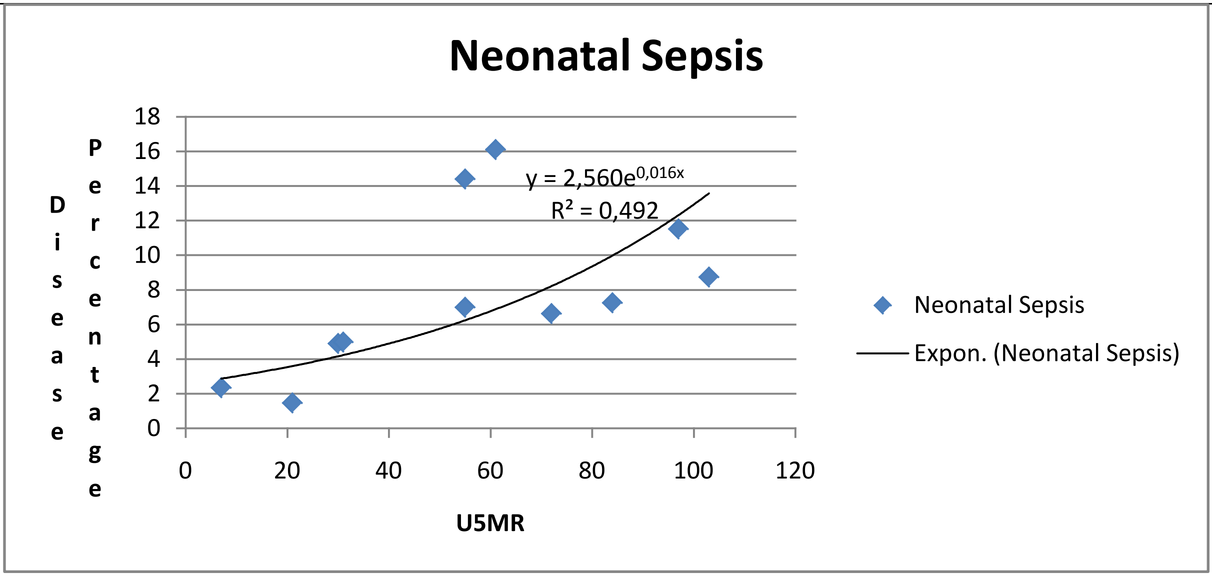
Neonatal Sepsis
Disease Percentage
18
16
14
12
10
8
6
4
2
0
0
20
40
60
80
100
120
U5MR
y = 2,560e^0,016x
R² = 0,492
Neonatal Sepsis
Expon. (Neonatal Sepsis)

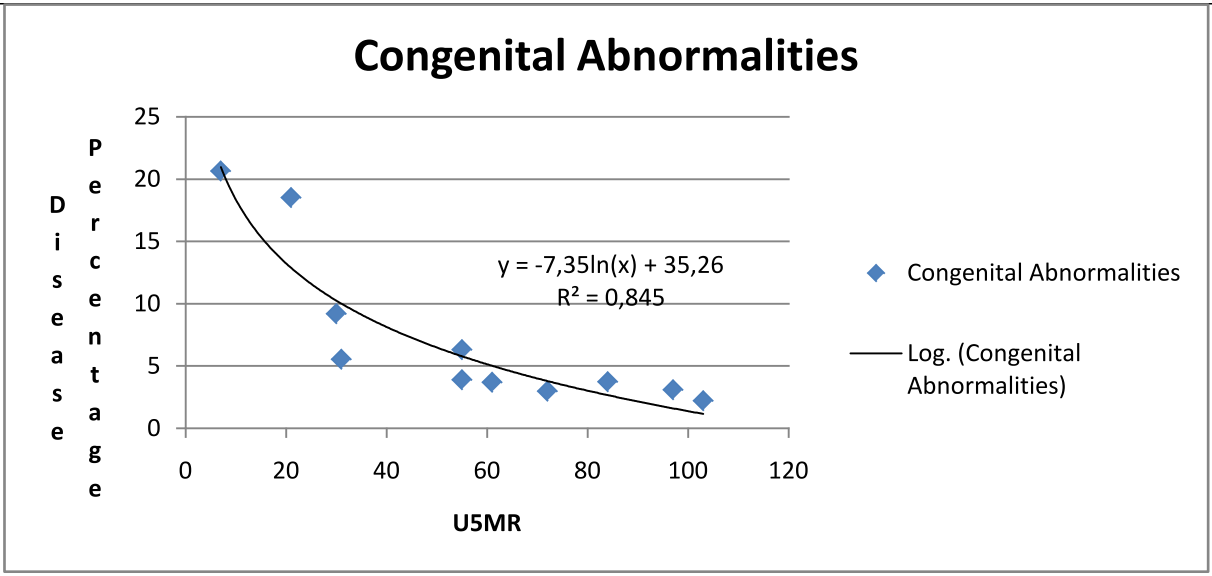
Congenital Abnormalities
Disease Percentage
25
20
15
10
5
0
0
20
40
60
80
100
120
U5MR
y = -7,35ln(x) + 35,26
R² = 0,845
Congenital Abnormalities
Log. (Congenital Abnormalities)

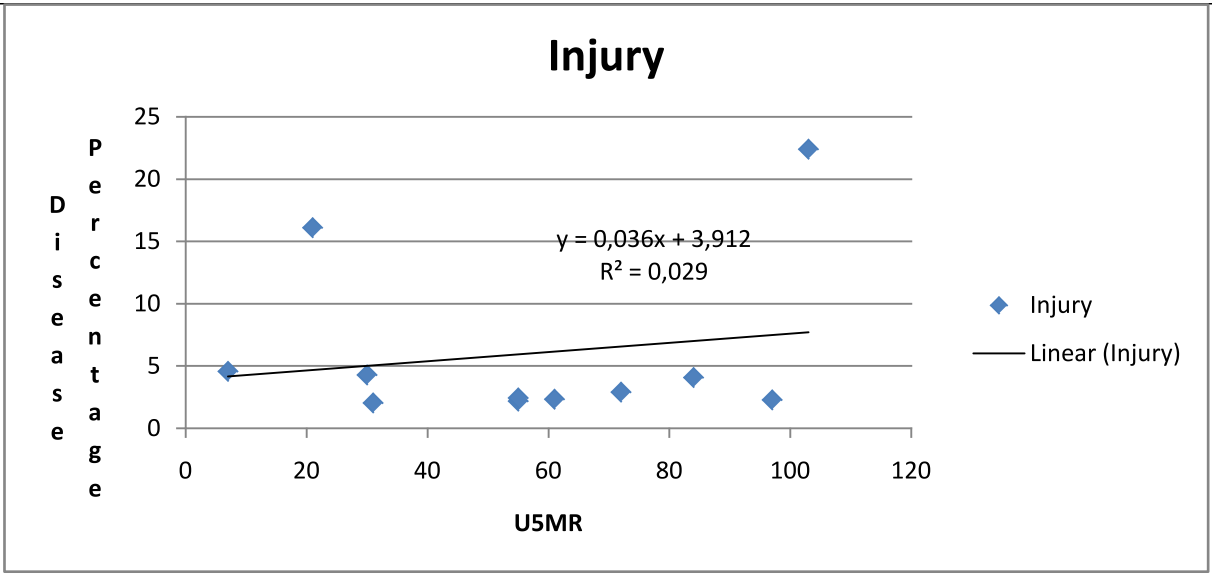
Injury
Disease Percentage
25
20
15
10
5
0
0
20
40
60
80
100
120
U5MR
y = 0,036x + 3,912
R² = 0,029
Injury
Linear (Injury)

5. Americas

		Relative Disease Proportions								
**Country**	**U5MR**	**AIDS**	**Diarrhoea**	**Malaria**	**Pneumonia**	**Preterm birth complications**	**Birth asphyxia**	**Neonatal Sepsis**	**Congenital Abnormalities**	**Injury**
Argentina	16.00	0.15	0.90	0.00	7.11	29.57	5.49	5.67	2572.00	696.00
Barbados	12.00	3.03	0.00	0.00	3.03	36.36	15.15	6.06	4.00	2.00
Belize	25.00	4.17	7.64	0.00	11.11	18.06	9.03	3.47	21.00	15.00
Bolivia	57.00	0.20	15.23	0.02	18.22	15.86	12.59	8.25	650.00	436.00
Brazil	22.00	0.23	5.32	0.06	8.77	23.07	10.08	6.57	9754.00	3403.00
Canada	6.00	0.00	0.05	0.00	1.36	27.33	12.08	1.86	541.00	128.00
Chile	9.00	0.13	0.27	0.00	5.60	24.52	4.75	2.82	785.00	133.00
Colombia	20.00	0.28	4.14	0.07	12.24	19.28	7.09	6.92	3136.00	1300.00
Costa Rica	11.00	0.00	1.42	0.00	5.03	22.97	9.29	5.55	236.00	26.00
Cuba	7.00	0.13	1.33	0.00	10.80	9.60	6.80	8.67	183.00	57.00
Dominican Republic	38.00	1.31	9.22	0.05	18.42	25.65	11.31	4.38	683.00	204.00
Ecuador	22.00	0.91	5.53	0.00	17.06	21.77	8.80	1.65	1095.00	443.00
El Salvador	24.00	3.90	3.63	0.00	13.68	21.03	8.85	1.15	420.00	149.00
Guatemala	39.00	2.77	19.07	0.00	20.22	18.58	4.64	0.94	797.00	769.00
Guyana	60.00	5.80	15.24	0.85	17.17	15.84	9.43	6.53	67.00	37.00
Haiti	76.00	4.55	20.28	0.87	20.15	11.00	9.95	5.87	459.00	462.00
Honduras	24.00	1.37	9.85	0.03	17.64	21.53	11.72	5.29	570.00	241.00
Jamaica	31.00	3.69	13.99	0.00	22.65	13.39	5.27	0.91	156.00	81.00
Mexico	35.00	0.23	5.50	0.00	12.51	17.45	6.82	4.87	7989.00	3200.00
Nicaragua	35.00	0.65	8.92	0.00	19.69	21.87	8.40	1.61	495.00	173.00

Panama	23.00	0.68	6.03	0.00	13.35	15.81	6.46	5.23	390.00	105.00
Paraguay	29.00	1.00	8.76	0.00	16.80	24.08	12.65	4.57	442.00	166.00
Peru	20.00	1.29	4.46	0.12	15.51	24.72	9.87	1.85	2319.00	920.00
Suriname	29.00	2.33	5.84	0.78	10.12	22.18	10.12	5.84	31.00	20.00
Trinidad and Tobago	35.00	6.17	0.57	0.00	5.74	21.66	6.60	7.17	144.00	63.00
Uruguay	14.00	0.13	2.01	0.00	7.14	20.05	4.26	5.64	220.00	86.00
USA	8.00	0.02	0.09	0.00	2.53	30.05	4.79	2.59	8001.00	4013.00
Venezuela	19.00	0.30	7.19	0.01	10.35	23.24	8.65	8.04	2035.00	1019.00

Models for disease proportion against U5MR

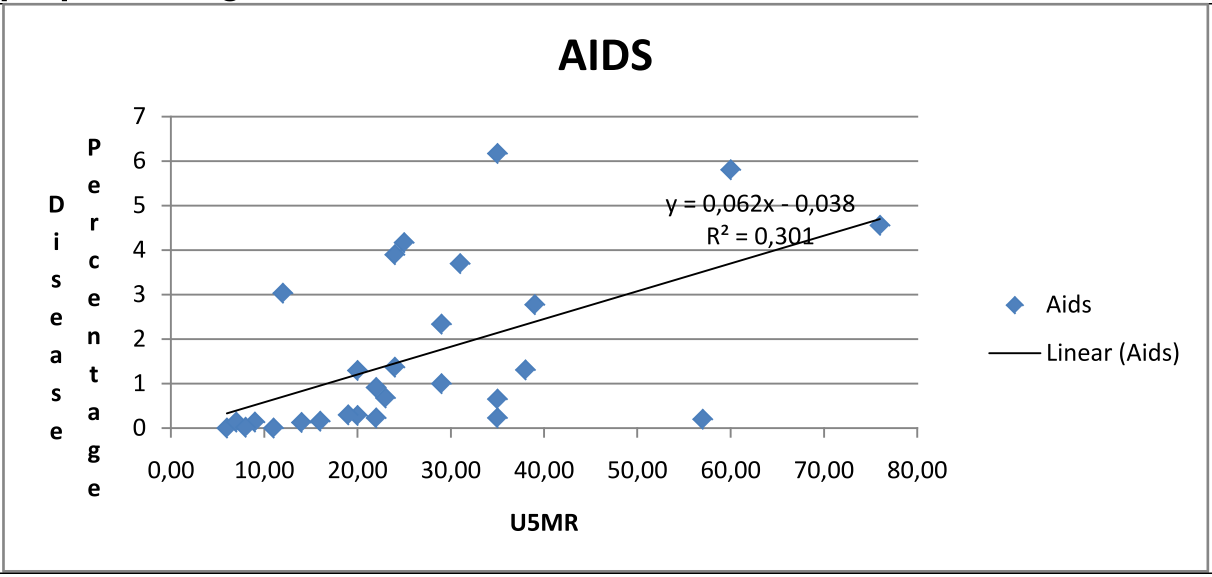

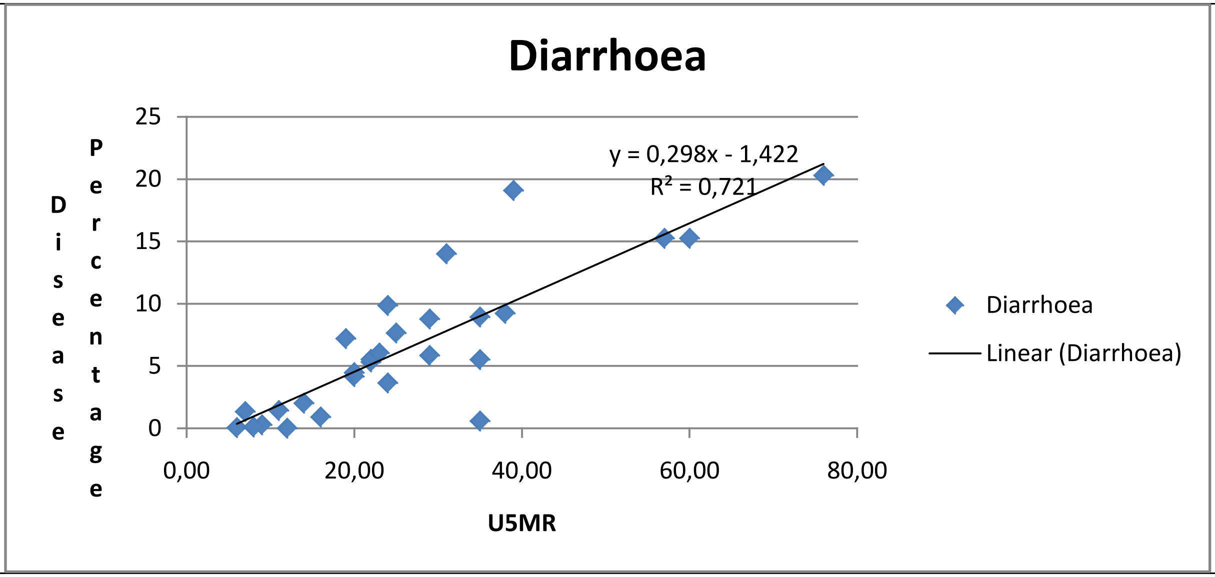

Diarrhoea
y = 0,298x - 1,422
R² = 0,721
25
20
15
10
5
0
0,00
20,00
40,00
60,00
80,00
U5MR
Disease Percentage
Diarrhoea
Linear (Diarrhoea)

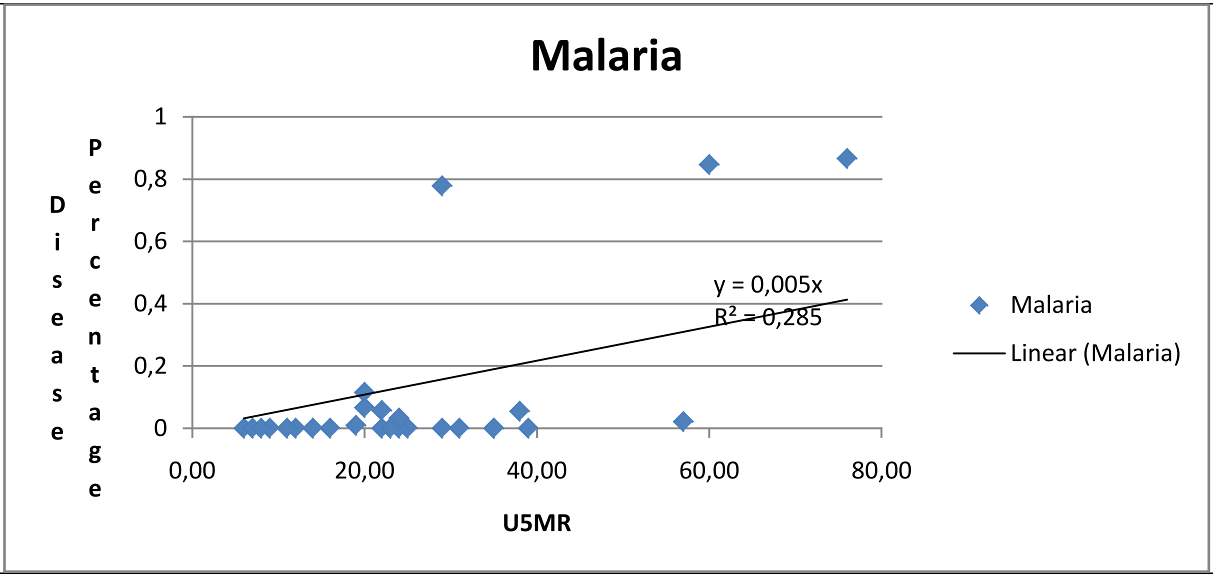

Malaria
1
0,8
0,6
0,4
0,2
0
Disease Percentage
0,00
20,00
40,00
60,00
80,00
U5MR
y = 0,005x
R² = 0,285
Malaria
Linear (Malaria)

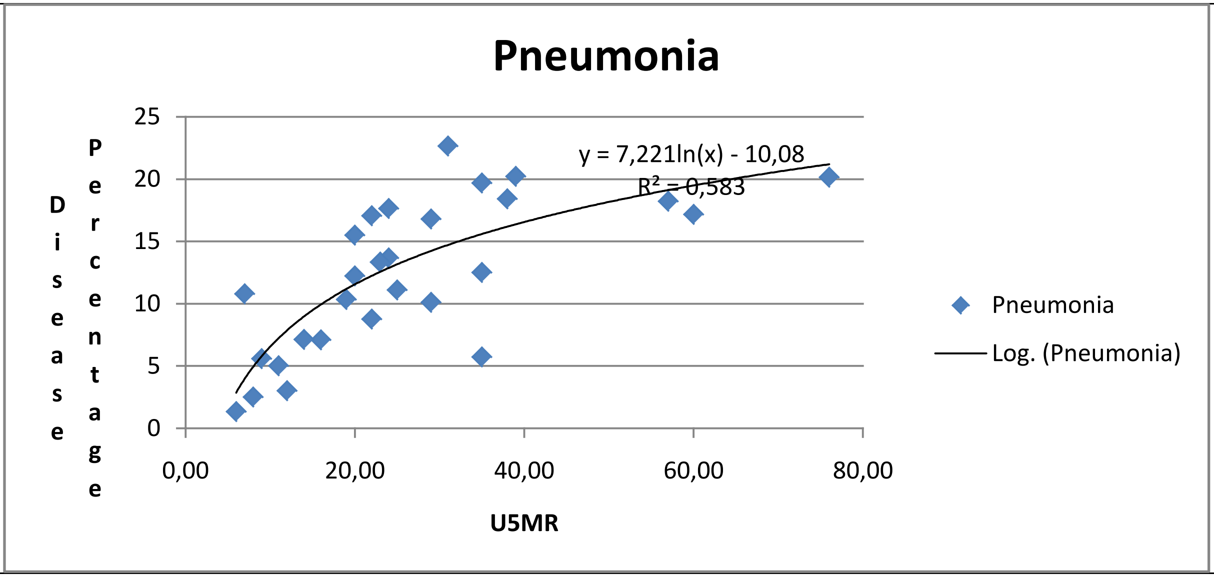
Pneumonia
Disease Percentage
25
20
15
10
5
0
0,00
20,00
40,00
60,00
80,00
U5MR
y = 7,221ln(x) - 10,08
R² = 0,583
Pneumonia
Log. (Pneumonia)

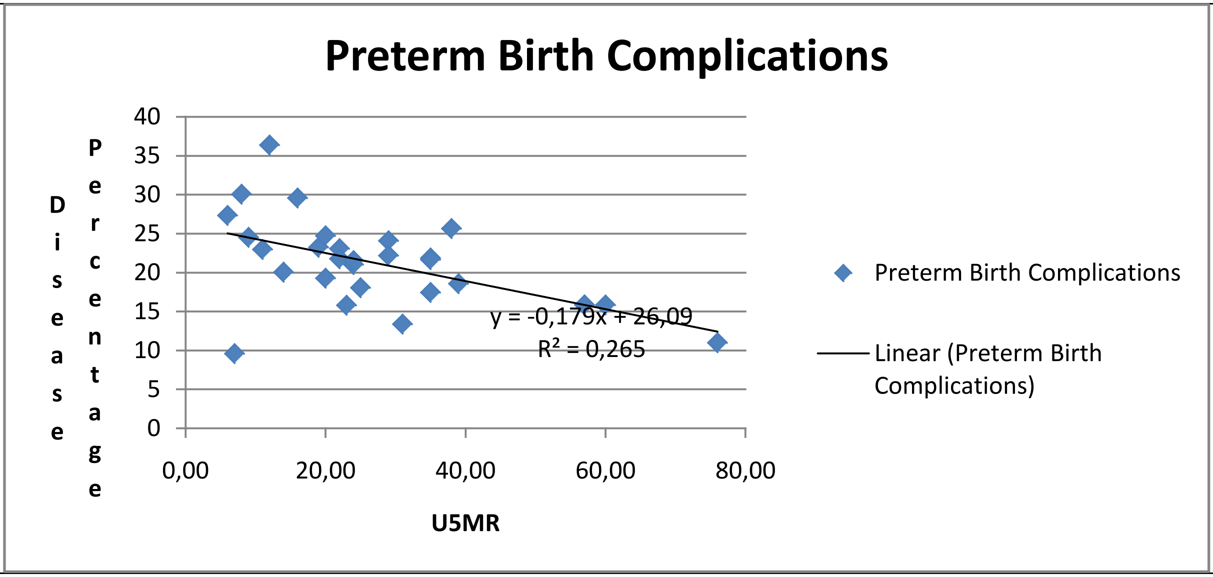

Preterm Birth Complications
Disease
Percentage
40
35
30
25
20
15
10
5
0
0,00
20,00
40,00
60,00
80,00
U5MR
y = -0,179x + 26,09
R² = 0,265
Preterm Birth Complications
Linear (Preterm Birth Complications)

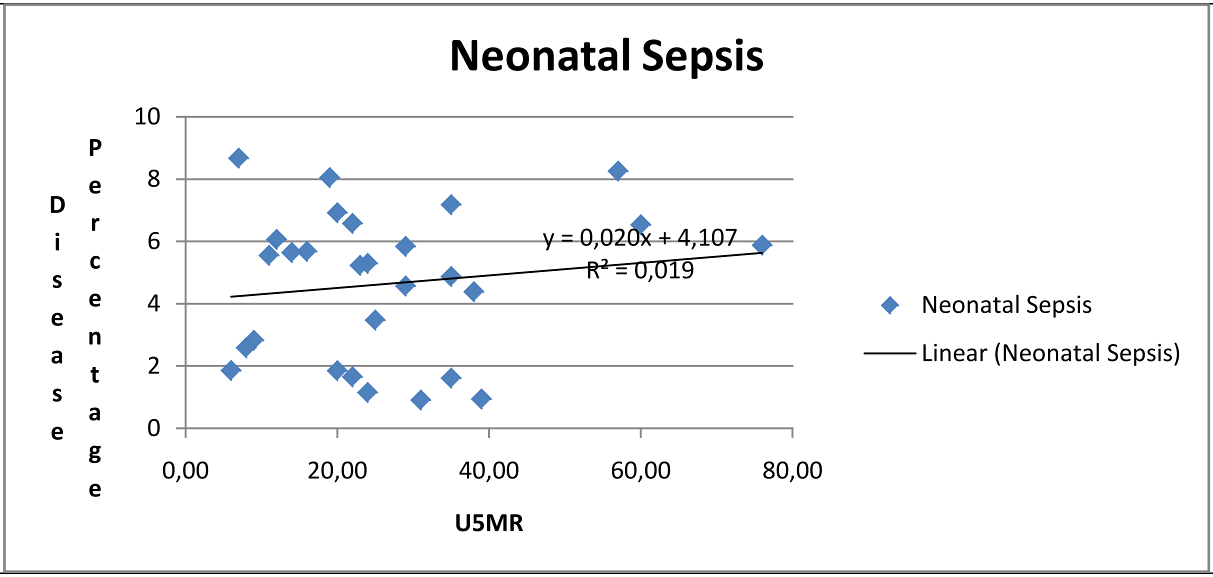
Neonatal Sepsis
Disease Percentage
10
8
6
4
2
0
0,00
20,00
40,00
60,00
80,00
U5MR
y = 0,020x + 4,107
R² = 0,019
Neonatal Sepsis
Linear (Neonatal Sepsis)

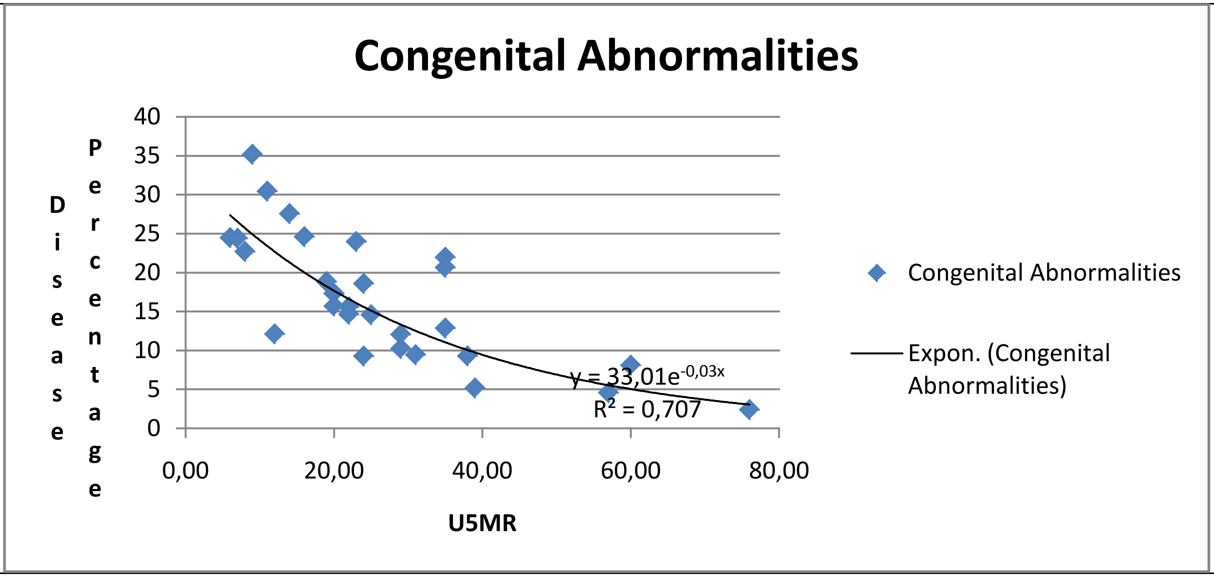
Congenital Abnormalities
Disease Percentage
40
35
30
25
20
15
10
5
0
0,00
20,00
40,00
60,00
80,00
U5MR
y = 33,01e^-0,03x
R² = 0,707
Congenital Abnormalities
Expon. (Congenital Abnormalities)

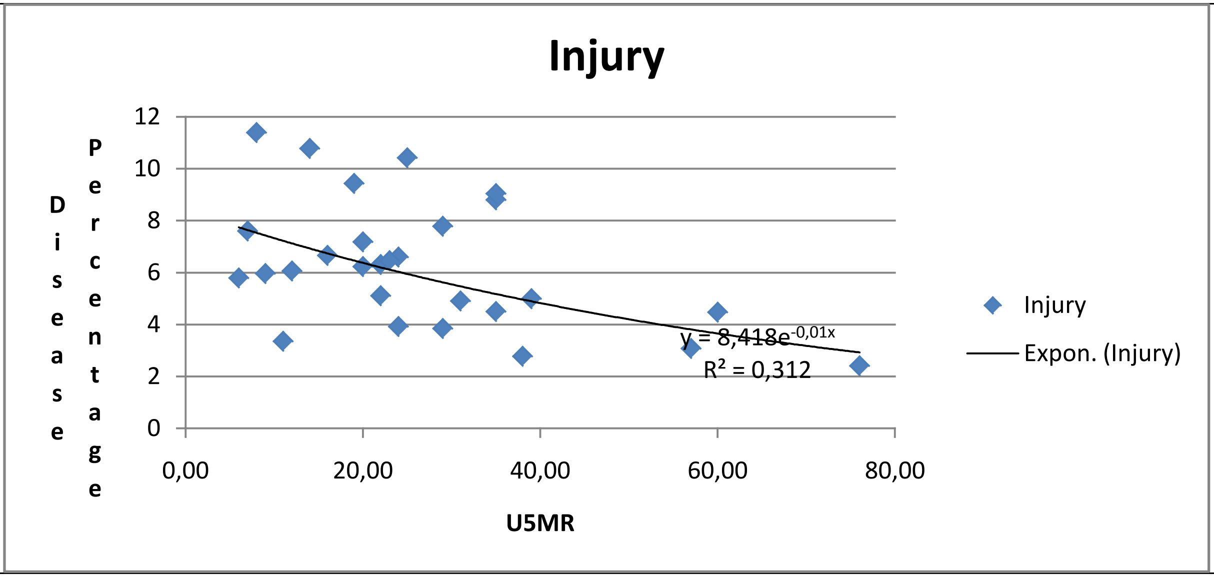

Injury
Disease Percentage
12
10
8
6
4
2
0
0,00
20,00
40,00
60,00
80,00
U5MR
y = 8,418e-0,01x
R² = 0,312
Injury
Expon. (Injury)

Table w3. Quintile-specific numbers of deaths in 2008 from each cause of death in the five exemplar countries.

Nigeria

			Number of Under 5 Deaths in 2008									
Quintile	U5MR	Total Number of Under 5 Deaths in 2008	AIDS	Diarrhoea	Malaria	Pneumonia	Preterm birth complications	Birth asphyxia	Neonatal Sepsis	Congenital Abnormalities	Injury	Other*
Q1 (wealthiest)	219.00	103686.60	8522.21	12916.86	10316.33	15282.55	13599.61	9186.78	5386.62	5062.79	5062.79	18350.07
Q2	212.00	153742.20	9640.25	23162.49	22108.39	24749.77	15740.45	12073.87	7965.85	4437.72	4437.72	29425.69
Q3	165.00	196647.00	9045.76	34022.68	33722.35	34142.71	16596.93	14206.18	10171.96	3223.18	3223.18	38292.06
Q4	129.00	252661.60	6112.39	51088.43	50451.17	48418.88	16697.85	16634.14	13047.34	931.87	931.87	48347.65
Q5 (Poorest)	87.00	261004.20	5466.47	53909.90	53070.71	50758.31	16629.73	16966.66	13475.20	532.93	532.93	49661.36

Egypt

			Number of Under 5 Deaths in 2008									
Quintile	U5MR	Total Number of Under 5 Deaths in 2008	AIDS	Diarrhoea	Malaria	Pneumonia	Preterm birth complications	Birth asphyxia	Neonatal Sepsis	Congenital Abnormalities	Injury	Other *
Q1 (wealthiest)	49.00	6955.20	10.37	301.08	22.78	624.21	1993.41	455.89	144.99	1435.03	456.11	1511.32
Q2	36.10	10009.60	36.76	717.13	107.55	1116.16	2684.52	718.12	276.33	1853.99	582.81	1916.23
Q3	32.20	11849.60	55.50	1004.70	168.34	1440.86	3053.39	891.21	364.26	2056.68	649.55	2165.11
Q4	27.20	13284.80	71.32	1244.69	219.89	1706.16	3318.05	1033.23	436.58	2191.79	697.54	2365.54
Q5 (Poorest)	18.90	18032.00	129.81	2118.58	411.48	2645.16	4062.12	1539.82	694.90	2515.69	835.52	3078.91

Bangladesh

			Number of Under 5 Deaths in 2008									
Quintile	U5MR	Total Number of Under 5 Deaths in 2008	AIDS	Diarrhoea	Malaria	Pneumonia	Preterm birth complications	Birth asphyxia	Neonatal Sepsis	Congenital Abnormalities	Injury	Other *
Q1 (Wealthiest)	86.00	34382.80	141.07	3679.03	284.86	5461.23	5935.23	4420.67	1766.56	2612.19	1889.30	8192.67
Q2	85.00	49575.20	118.86	6141.44	908.07	8237.76	7317.81	6776.19	3465.19	2432.12	3070.74	11107.03
Q3	83.00	66366.80	68.90	9114.58	1951.52	11415.80	8473.20	8997.48	6518.69	1832.01	4623.71	13370.92
Q4	62.00	67966.00	63.02	9408.85	2070.31	11723.29	8566.76	9169.56	6895.60	1757.13	4785.15	13526.32
Q5 (Poorest)	43.00	68765.60	60.01	9556.65	2130.98	11877.33	8612.57	9252.34	7090.67	1718.64	4866.75	13599.66

Cambodia

			Number of Under 5 Deaths in 2008									
Quintile	U5MR	Total Number of Under 5 Deaths in 2008	AIDS	Diarrhoea	Malaria	Pneumonia	Preterm birth complications	Birth asphyxia	Neonatal Sepsis	Congenital Abnormalities	Injury	Other *
Q1 (wealthiest)	90.00	2292.00	11.72	123.82	19.80	449.04	422.74	191.89	58.25	0.00	134.95	879.79
Q2	83.00	3743.60	17.30	252.16	52.83	880.76	599.50	330.13	123.60	263.19	165.75	1058.37
Q3	68.00	5195.20	21.44	396.21	101.74	1358.85	705.71	466.34	211.01	210.52	172.98	1550.41
Q4	49.00	6341.20	23.70	517.97	151.58	1759.99	739.72	564.16	295.60	166.32	168.60	1953.56
Q5 (Poorest)	30.00	6876.00	24.44	576.79	178.23	1953.08	740.55	604.96	339.78	147.21	164.59	2146.37

Peru												
			Number of Under 5 Deaths in 2008									
Quintile	U5MR	Total Number of Under 5 Deaths in 2008	AIDS	Diarrhoea	Malaria	Pneumonia	Preterm birth complications	Birth asphyxia	Neonatal Sepsis	Congenital Abnormalities	Injury	Other *
**Q1 (wealthiest)**	9.00	1051.20	5.49	13.24	0.51	60.78	257.28	85.91	45.08	262.56	78.02	242.34
**Q2**	24.00	2803.20	40.82	174.31	3.63	360.61	610.48	236.25	128.65	439.80	168.64	640.01
**Q3**	24.00	2803.20	40.82	174.31	3.63	360.61	610.48	236.25	128.65	439.80	168.64	640.01
**Q4**	33.00	3854.40	77.74	329.56	6.87	584.48	777.03	333.23	183.87	457.49	204.42	899.69
**Q5 (Poorest)**	59.00	6891.20	250.62	1053.45	21.96	1334.12	1067.09	657.95	364.75	365.33	253.97	1521.97

* Includes Measles, Meningitis, Pertussis, Tetanus, Other infections and Other non-communicable diseases

Table w4. Case Fatality Rates models

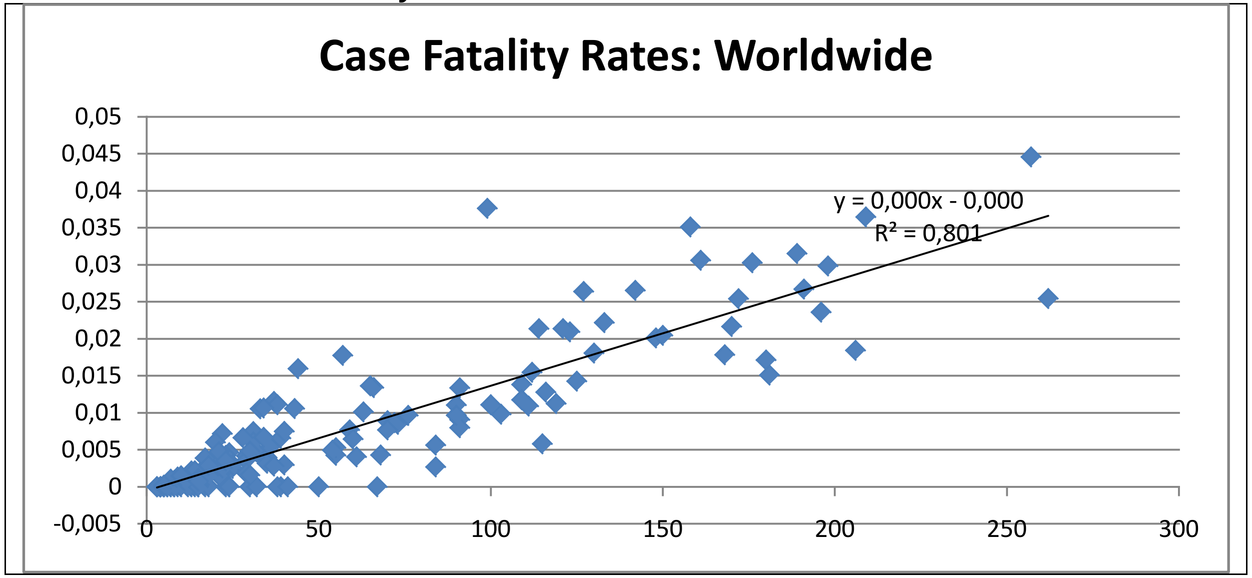

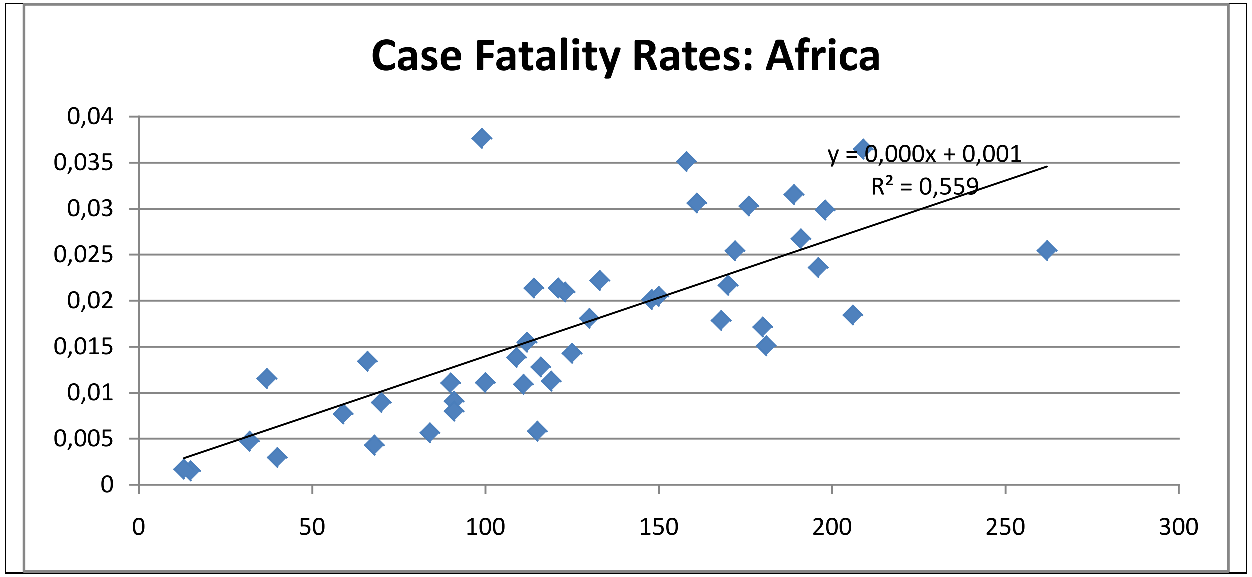
Case Fatality Rates: Africa
0,04
0,035
0,03
0,025
0,02
0,015
0,01
0,005
0
0
50
100
150
200
250
300
y = 0,000x + 0,001
R² = 0,559

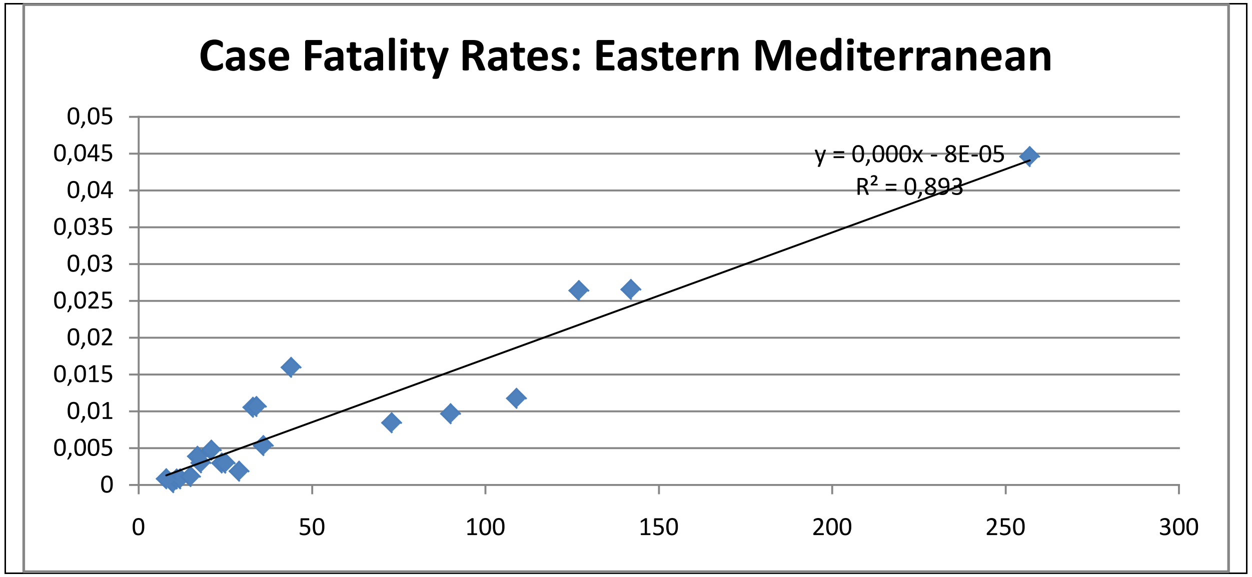
Case Fatality Rates: Eastern Mediterranean
y = 0,000x - 8E-05
R² = 0,893
0,05
0,045
0,04
0,035
0,03
0,025
0,02
0,015
0,01
0,005
0
0
50
100
150
200
250
300

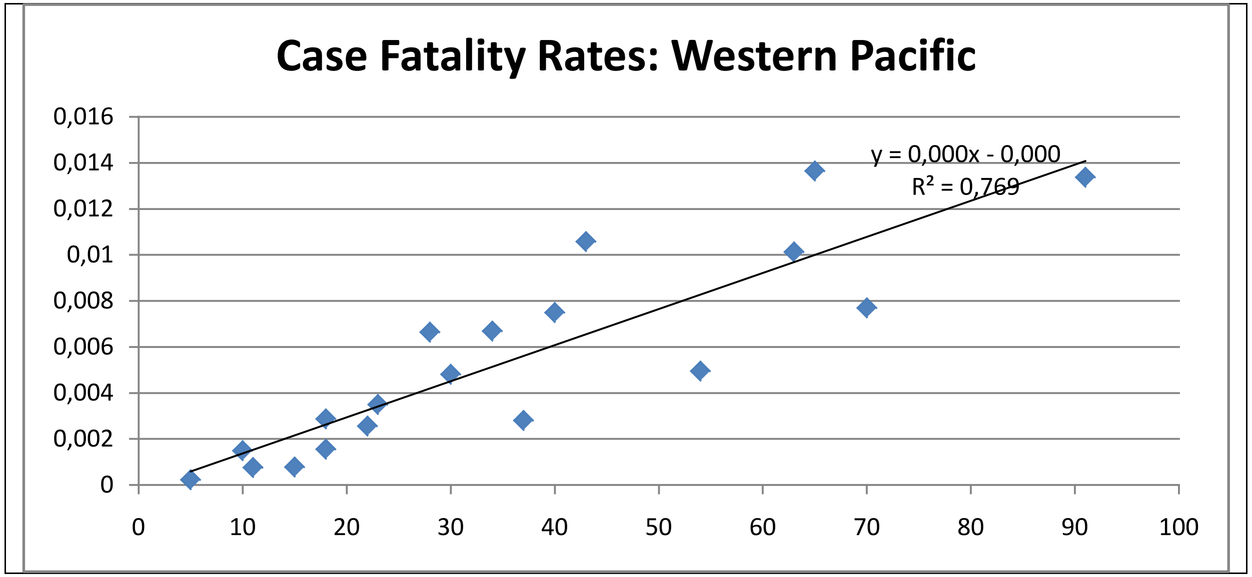
Case Fatality Rates: Western Pacific
0,016
0,014
0,012
0,01
0,008
0,006
0,004
0,002
0
0
10
20
30
40
50
60
70
80
90
100
y = 0,000x - 0,000
R² = 0,769

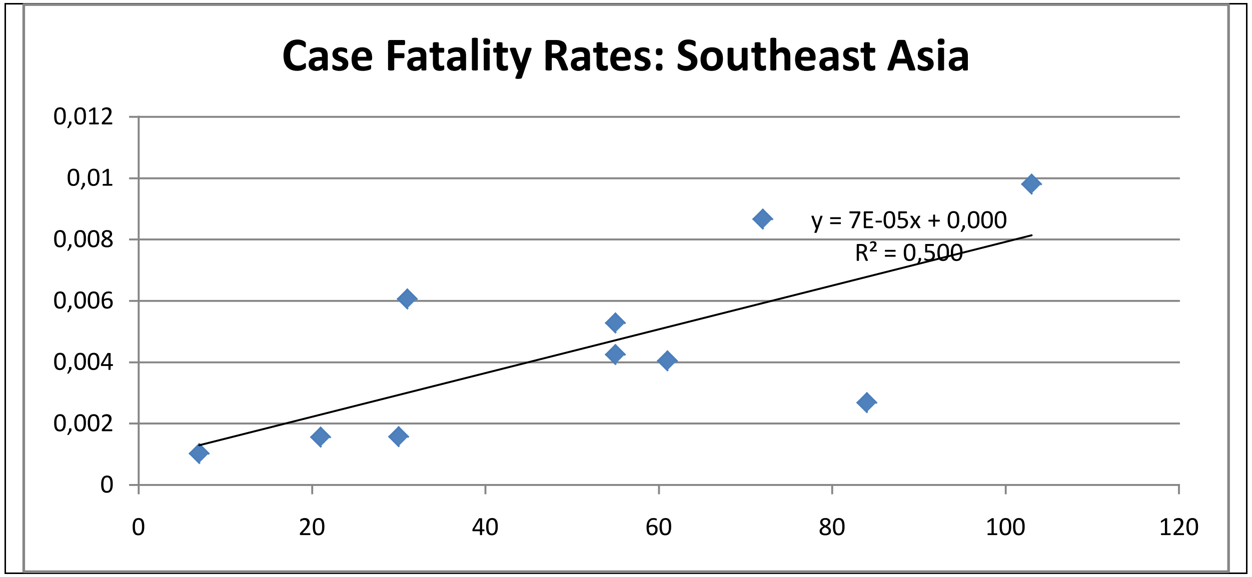
Case Fatality Rates: Southeast Asia
0,012
0,01
0,008
0,006
0,004
0,002
0
0
20
40
60
80
100
120
y = 7E-05x + 0,000
R² = 0,500

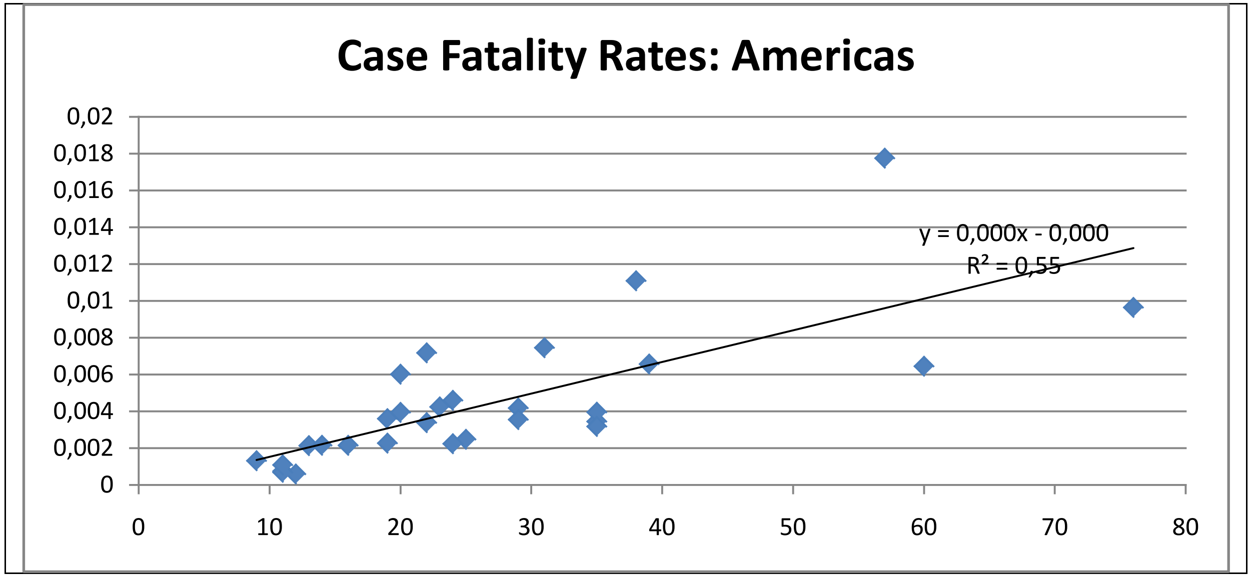
Case Fatality Rates: Americas
0,02
0,018
0,016
0,014
0,012
0,01
0,008
0,006
0,004
0,002
0
0
10
20
30
40
50
60
70
80
y = 0,000x - 0,000
R² = 0,55
